# Interleukin-17A signaling promotes CD8^+^ T cell cytotoxicity against West Nile virus infection through enhancing PI3K-mTOR-mediated metabolism

**DOI:** 10.1371/journal.ppat.1013218

**Published:** 2025-07-09

**Authors:** Farzana Nazneen, Biswas Neupane, Yao Chen, Shazeed-Ul Karim, Zongbing You, Weiguo Cui, Fengwei Bai

**Affiliations:** 1 Cell and Molecular Biology Program, School of Biological, Environmental, and Earth Sciences, The University of Southern Mississippi, Hattiesburg, Mississippi, United States of America; 2 Department of Microbiology and Immunology, Medical College of Wisconsin, Milwaukee, Wisconsin, United States of America; 3 Department of Structural & Cellular Biology, Tulane University, New Orleans, Louisiana, United States of America; 4 Department of Pathology, Northwestern University, Chicago, Illinois, United States of America; Princeton University, UNITED STATES OF AMERICA

## Abstract

West Nile Virus (WNV), a mosquito-borne neurotropic flavivirus, is a major cause of viral encephalitis in the United States, posing a continuous threat to public health. Unfortunately, no vaccine or specific therapeutic intervention is available against WNV infection. Previous studies, including ours, demonstrated that interleukin-17A (IL-17A) signaling promotes the cytotoxicity of CD8^+^ T cells to facilitate WNV and parasite clearance; however, the molecular mechanism is not understood. IL-17 receptor C (IL-17RC) is an obligatory co-receptor with IL-17 receptor A (IL-17RA) for signaling induced by IL-17A, IL-17A/F, and IL-17F. In this study, we found that IL-17RC deficient (*Il17rc*^*-/-*^) mice were more susceptible to WNV infection with a higher viral load in the brain than wild-type (WT) control mice. The number of infiltrating WNV-specific CD8^+^ T cells and the expression levels of cytotoxicity mediators, such as perforin, in the T cells in the brain of *Il17rc*^*-/-*^ mice were reduced. In addition, WNV-specific CD8^+^ T cells from IL-17RA deficient (*Il17ra*^*-/-*^) mice and CD8^+^ cell-specific *Il17ra* conditional knockout (cre-KO) mice expressed lower levels of perforin than their counterpart controls. Moreover, supplementing mouse recombinant IL-17A *ex vivo* increased the perforin production in WNV-specific CD8^+^ T cells from the WT mice but not *Il17rc*^*-/-*^ or cre-KO mice. Interestingly, we found that IL-17A signaling activated the phosphatidylinositol-3-kinase/mammalian target of rapamycin (PI3K-mTOR) signaling pathway in CD8^+^ T cells, leading to increased metabolism of CD8^+^ T cells to cope with the higher energy demand for WNV clearance in the brain. In summary, our findings reveal a novel IL-17A-PI3K-mTOR signaling axis in promoting the effector functions of CD8^+^ T cells, suggesting potential broader implications in stimulating immune responses to combat WNV and other intracellular infections.

## Author summary

West Nile encephalitis is the leading viral neurological disease in the United States and North America. No approved vaccine or specific therapeutics are available to prevent or treat human West Nile virus (WNV) infection. Interleukin 17A (IL-17A) is a pleiotropic cytokine that regulates inflammatory diseases and cancers. One of our previous studies has demonstrated that IL-17A signaling promotes the cytotoxicity of CD8^+^ T cells and facilitates WNV clearance from the brain in the mouse model, as well as the therapeutic potential of this cytokine. However, the molecular mechanism is not understood. This study uncovered a new role of IL-17A signaling in activating a metabolic pathway in CD8^+^ T cells to cope with the higher energy demand for WNV clearance from the brain. This finding may have broader therapeutic implications in stimulating IL-17A-mediated metabolic pathways in CD8^+^ T cells to combat other viral and intracellular infections.

## Introduction

West Nile virus (WNV) is a mosquito-transmitted, single-stranded, positive-sense RNA virus belonging to the *Flaviviridae* family [1]. Human WNV infection is typically subclinical, but in symptomatic individuals, the manifestations range from fever and myalgias to meningoencephalitis [2–5]. The WNV-associated neuroinvasive disease affects all ages; however, elderly and immunocompromised individuals are particularly at risk [6], with 4–24% mortality rates among the elderly with encephalitis [7]. WNV was initially identified in the West Nile province of Uganda in 1937 [8], and since it arrived in the United States in 1999, WNV has claimed the lives of over 2,900 people and sickened more than 60,600 individuals as of January 14, 2025, as reported by the Centers for Disease Control and Prevention (CDC) [9,10]. Furthermore, up to 50% of WNV survivors experience significant morbidity for at least a year following the infection [11]. WNV has become the primary viral agent responsible for human neurological diseases in North America [12]. Despite the intensive research of over 20 years, vaccines or specific treatments are still unavailable for human WNV infection, and treatment remains supportive [1].

After an infected mosquito bite, WNV replicates in various cells, including keratinocytes, neutrophils, and monocytes, and disseminates via the bloodstream into peripheral organs, such as the liver, kidney, and spleen [1,13]. Although WNV induces a lower level of viremia that usually clears up within a few days in humans, it may invade the central nervous system (CNS) in some individuals, causing various neuroinvasive complications [14]. While innate immune responses serve as the first line of host defense in controlling the viral replication in the peripheral cells and organs, the B and T cell-mediated adaptive immunity plays a key role in clearing the WNV infection in the CNS. Particularly, CD8^+^ T cells are essential in protecting from WNV infection, and they control viral infections by triggering apoptosis of infected cells through perforin- or Fas ligand-dependent pathways or producing antiviral cytokines (e.g., IFN-γ and TNF-α). Our previous study demonstrated that interleukin-17A (IL-17A) enhanced CD8^+^ T cell cytotoxicity, and a supplement of IL-17A as late as day 6 post-WNV infection significantly increased the survival of the infected mice [15], suggesting a therapeutic role of IL-17A against WNV infection in the CNS.

IL-17A is the founding member of IL-17 family that also includes IL-17B, IL-17C, IL-17D, IL-17E (IL-25), and IL-17F [15,16]. With 50% structural homology to IL-17F, IL-17A acts as both a homodimer and an IL-17A/IL-17F heterodimer [15]. IL-17A is produced primarily by T helper 17 (Th17) cells, cytotoxic T 17 cells (Tc17), γδT cells, natural killer T (NKT) cells, group 3 innate lymphoid (ILC3) cells, neutrophils, and mast cells [15,17,18]. The biological functions of IL-17A and IL-17F are mediated through binding to IL-17 receptor A (IL-17RA) and IL-17 receptor C (IL-17RC) complex [19]. While IL-17RA participates in the formation of the receptor complexes for other members of the IL-17 family, IL-17RC is specific to IL-17A signaling [20]. IL-17RA is widely expressed in the bone marrow, thymus, spleen, and hematopoietic cells, whereas IL-17RC is more abundant in epithelial cells of prostate, kidney, and joints, with lower levels in hematopoietic cells [21–23].

IL-17A is a pro-inflammatory cytokine with prominent roles in allergic and autoimmune diseases, such as multiple sclerosis [24–28], rheumatoid arthritis [29–33], psoriasis [33–36], asthma [31,37,38], Crohn’s disease [39,40], and cancers [41–43]. IL-17A signaling mediates protective immunity against fungi and bacteria by promoting neutrophil recruitment, inducing antimicrobial peptides, and enhancing barrier functions [19]. In terms of viral infections, IL-17A plays both protective and pathogenic roles. It promotes infections in influenza virus [44], respiratory syncytial virus [45], murine encephalitis virus [46], hepatitis virus [47], and chikungunya virus (CHIKV) [48]. IL-17A can also promote the adaptive immune responses to lymphocytic choriomeningitis virus infection [49]. Research from our lab indicates that IL-17A enhances the cytotoxicity of CD8^+^ T cells, thereby promoting WNV clearance from the brain in a mouse model [50]. Consistent with our findings, IL-17A signaling has also been observed to sustain CD8^+^ T cell immunity against *Trypanosoma cruzi* infection [51] to enhance CD8^+^ T cell effector function [52]. During other viral infections, IL-17A was found to be crucial for priming hepatic CD8^+^ T cells in adenovirus-infected mice [53] and partly responsible for the clustering and activation of CD8^+^ T cells in the spleen following vaccinia virus infection [54]. However, the detailed mechanism by which IL-17A signaling enhances the cytotoxicity of CD8^+^ T cells is not understood. In this study, we unveiled a novel function of IL-17A, which shows that it activates the PI3K-mTOR signaling pathway-mediated metabolism in CD8^+^ T cells to cope with a higher energy demand and protein synthesis of the cytotoxicity mediators, such as perforin, for WNV clearance in the brain.

## Results

### IL-17RC is essential to protect mice from WNV infection

**Fig 1:**
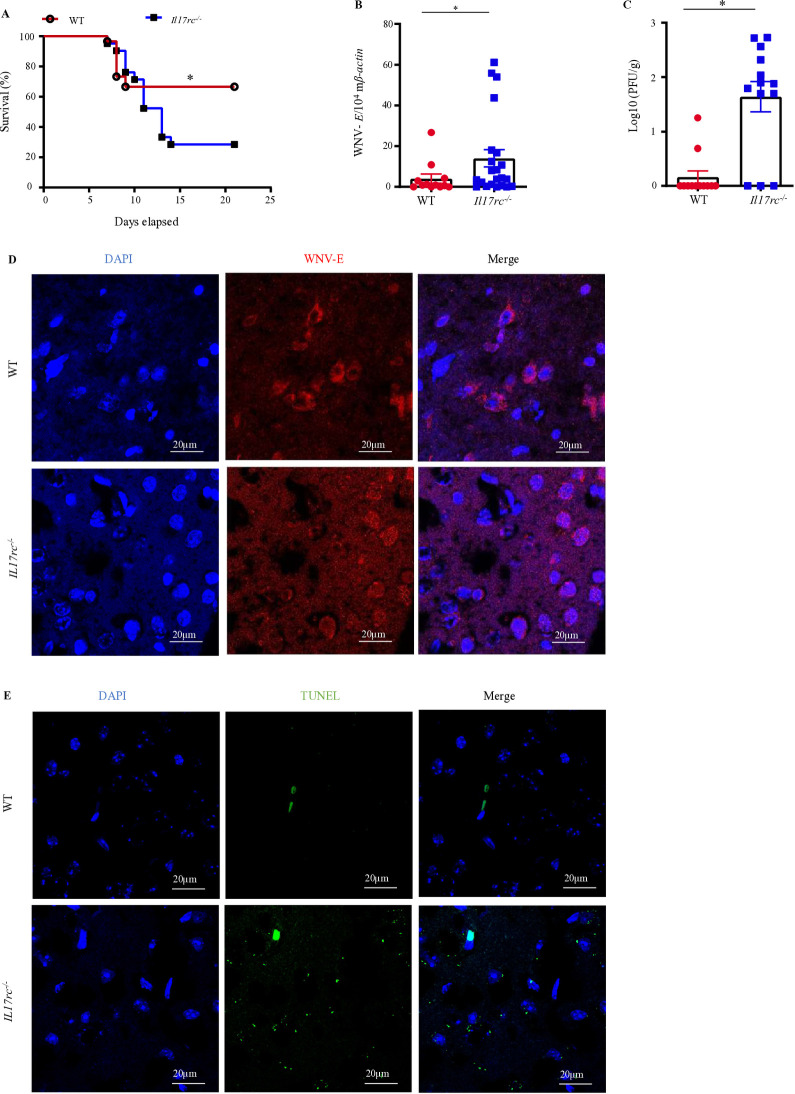
*Il17rc*^*-*^*/*^*-*^ mice are susceptible to WNV infection. Eight to nine weeks old *Il17rc*^*-/-*^ (n = 21) and WT mice (n = 30) were infected with 100 PFU of WNV via f.p. inoculation and monitored daily for mortality for 21 days. (A) The survival curves of *Il17rc*^*-/-*^ and WT mice after WNV infection. The survival was compared with a Log-rank test with * denoting *p* <0.05. Brain tissue was collected on D7 p.i., and the viral load was measured by qRT-PCR (B, WT, n =12; *Il17rc*^*-/-*^, n = 22), and plaque-forming assay (C, WT, n = 14; *Il17rc*^*-/-*^, n = 13). Data were presented as mean ± s.e.m. and compared with two-tailed student’s *t*-tests with * denoting *p* <0.05. The data represent at least three independent experiments. (D) IFA images of WNV-E antigens in mouse brains. The WNV-E antigen and nuclei were stained with Alexa fluor 568 (red) and DAPI (blue). (E) TUNEL assay images of WNV-infected mouse brain. The TUNEL-positive cells and nuclei were stained with FITC (green) and DAPI (blue). Images in (D) and (E) were taken by Nikon A1R confocal microscope at 40x magnification (2.5x zoomed in, scale bar = 20 µm).

It has been reported that IL-17A initiates its signaling via the IL-17RA/IL-17RC receptor complex [20]. While some other members of the IL-17 cytokine family use IL-17RA as one of their receptor subunits, IL-17RC is an obligate co-receptor with IL-17RA for signaling induced by IL-17A [20]. Therefore, we employed the *Il17rc*^*-/-*^mice [55] to study the signaling of IL-17A in the pathogenesis of WNV. Eight to nine weeks old *Il17rc*^*-/-*^ and WT (C57BL/6J) mice were infected with 100 plaque-forming units (PFU) of WNV via the footpad (f.p.) route, which partially mimicked mosquito inoculation, and monitored the survival for 21 days. The infected animals showed a wide range of disease symptoms, such as ruffled fur, hunched back, increased body temperature, weight loss, some form of paralysis, mostly hind limb weakness, decreased movement, huddled together, and increased mortality [56]. The survival results showed a lower survival rate in *Il17rc*^*-/-*^ (28%) than in WT (66%) mice (Fig 1A), suggesting that the absence of the IL-17RC results in *Il17rc*^*-/-*^ mice being more susceptible to WNV infection. After WNV infection in mice, the viral burden in the blood and peripheral organs typically peaks between days 3 and 4 post-infection (p.i.). Subsequently, the virus enters the central nervous system (CNS) and clears from the peripheral organs after about a week [57–59]. To monitor the virological profiles in *Il17rc*^*-/-*^ mice, blood, liver, and spleen samples were collected on days (D) 2, 4, and 6 p.i., and the viral load was measured by quantifying the WNV envelope gene (*WNV-E*) by RT-qPCR. There was no apparent difference in the blood, liver, and spleen samples between *Il17rc*^*-/-*^ and WT mice (S1A-C Fig). However, there was a significantly higher viral load in the brain tissues of *Il17rc*^*-/-*^ mice collected on D7 p.i. than in the WT controls by both RT-qPCR (Fig 1B) and plaque-forming assay (Fig 1C). To visualize the impact of WNV infection in the brains of *Il17rc*^*-/-*^ mice, the expression of WNV-E antigen was detected using an immunofluorescence assay (IFA). A higher expression of WNV-E antigen was observed in *Il17rc*^*-/-*^ mice (Fig 1D). In addition, TUNEL assay results show that there are more apoptotic bodies in the brains of *Il17rc*^*-/-*^ mice (Fig 1E).

**Fig 2:**
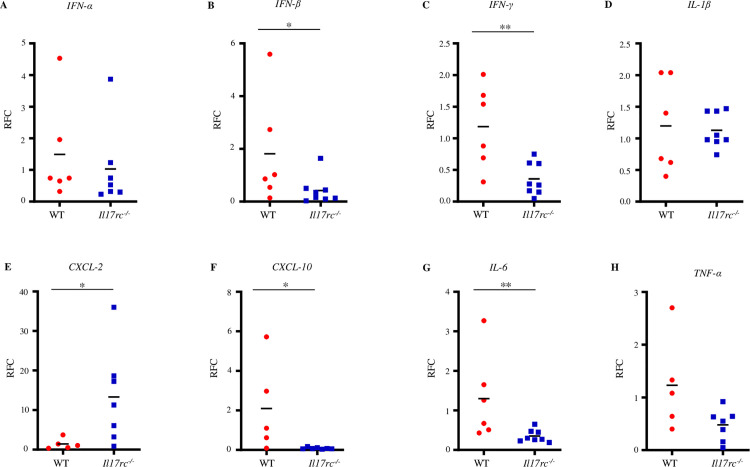
The antiviral and inflammatory responses in the brain of *Il17rc*^*-*^*/*^*-*^ mice. Eight to nine weeks old *Il17rc*^*-/-*^ (n = 8) and WT mice (n = 6) were infected with 100 PFU of WNV via f.p. inoculation, and brain tissues were collected on D7 p.i. and the expression of anti-viral and inflammatory cytokine genes were measured by RT-qPCR and normalized to mouse *β-actin*. The relative fold changes (RFC) of gene expressions of *IFN-α* (A), *IFN-β* (B), *IFN-γ* (C), *IL-1β* (D), *CXCL-2* (E), *CXCL-10* (F), *IL-6* (G), and *TNF-α* (H). Data were obtained from two independent experiments and presented as mean ± s.e.m. and analyzed by two-tailed student’s *t*-tests with *, and ** denoting *p* <0.05, and *p* <0.01, respectively.

Next, we evaluated the expression of inflammatory and antiviral cytokines and chemokines in the blood and brain tissues collected from *Il17rc*^*-/-*^ mice on D2 and D7 p.i., respectively. The RT-qPCR results showed no change in the transcripts of *IFN-α* (Fig 2A), *IL-1β* (Fig 2D), and *TNF-α* (Fig 2H). But interestingly, there was significant down-regulation in the transcripts of *IFN-β* (Fig 2B), *IFN-γ* (Fig 2C)*, CXCL-10* (Fig 2F)*,* and *IL-6* (Fig 2G) in the brain of *Il17rc*^*-/-*^ mice compared to WT controls. On the contrary, the expression of *CXCL-2* was increased in the brain of *Il17rc*^*-/-*^ mice compared to WT mice (Fig 2E). Compared to the brain samples, the changes of expression of these genes in the blood were mostly insignificant except for *IL-1β,* which was reduced in *Il17rc*^*-/-*^ mice (S2D Fig). Collectively, these findings suggest that *Il17rc*^*-/-*^ mice are more susceptible to WNV infection and IL-17A signaling via IL-17RC may play a more vital role in promoting the anti-viral function in brain than in the periphery to inhibit WNV infection.

### IL-17A-IL-17RC signaling promotes infiltration and cytotoxicity of CD8^+^ T cells in the brain

**Fig 3:**
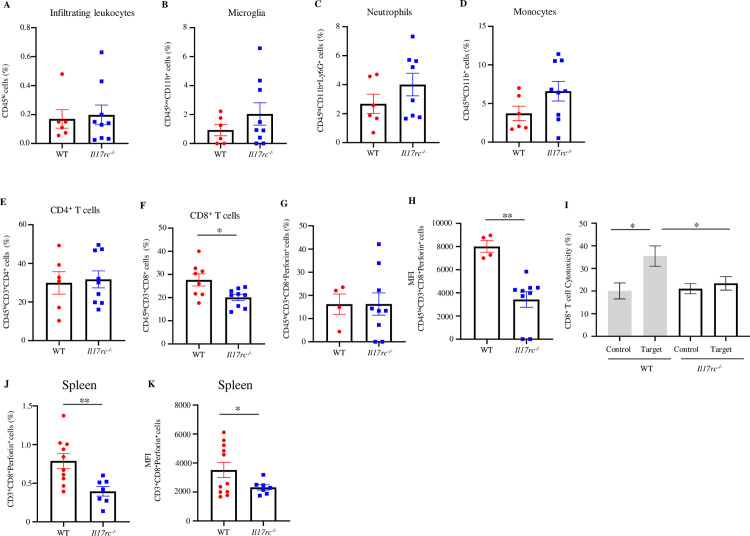
The cytotoxicity of infiltrated CD8^+^ T cells in the brain of *Il17rc*^*-*^*/*^*-*^ mice is reduced. Eight to nine weeks old *Il17rc*^*-/-*^ (n = 9) and WT mice (n = 4 to 10) were infected with 100 PFU of WNV via f.p. inoculation, and brain tissue was collected on D7 p.i. The brain leukocytes were characterized by flow cytometry after probing with antibodies against CD45, CD11b, Ly6G, CD3, CD4, CD8, and perforin. (A) The infiltrating leukocytes (CD45^hi^) in the brain of *Il17rc*^*-/-*^ and WT mice; (B) microglia (CD45^low^CD11b^+^); (C) neutrophils (CD45^hi^CD11b^+^Ly6G^+^); (D) monocytes (CD45^hi^CD11b^+^); (E) CD4^+^ T cells (CD45^hi^CD3^+^CD4^+^); (F) CD8^+^ T cells (CD45^hi^CD3^+^CD8^+^); (G) percentages of perforin expression in CD8^+^ T cells (CD45^hi^CD3^+^CD8^+^perforin^+^); (H) MFI of perforin expression in CD8^+^ T cells (CD45^hi^CD3^+^CD8^+^perforin^+^); and (I) CD8^+^ T cells isolated from leukocytes infiltrated into brain of *Il17rc*^*-/-*^ (n = 10) and WT mice (n = 10) were co-cultured with target (MC57GL_WNV-E_) or control (MC57GL_vector_) cells at a 10:1 effector/target ratio for 8 h, and cytotoxicity was evaluated by quantifying by the release of intracellular lactate dehydrogenase in culture supernatants. (J) percentages of perforin expression in splenic CD8^+^ T cells (CD3^+^CD8^+^perforin^+^); (K) MFI of perforin expression in splenic CD8^+^ T cells (CD3^+^CD8^+^perforin^+^); All experiments were performed at least twice. Data were presented as mean ± s.e.m. and analyzed by Mann-Whitney U tests (A-H) or two-tailed student’s *t*-tests (I-K) with * and ** denoting *p* < 0.05 and *p* < 0.01, respectively.

IL-17A signaling has been reported to facilitate the migration and activation of innate immune cells, such as neutrophils, during infections and autoimmune disorders [21,60]. Therefore, we asked if the absence of IL-17A signaling is linked to a deficiency of immune cell infiltration in responding to WNV infection in the brain. To address this question, *Il17rc*^*-/-*^ and WT mice were infected with WNV and sacrificed on D7 p.i. to collect the brain. Total leukocytes in the brain were isolated and characterized using flow cytometry. The results showed no change in the total infiltrating leukocytes in the brain of *Il17rc*^*-/-*^ mice compared to WT controls (Fig 3A). While no apparent changes were detected in the cell populations of residential microglia, infiltrating neutrophils, monocytes, or CD4^+^ T lymphocytes, a significant decrease in CD8^+^ T cell population was noted in *Il17rc*^*-/-*^ mice (Fig 3B-F), suggesting that IL-17A-IL-17RC signaling may promote the infiltration of CD8^+^ T cells into the brain during WNV infection. To exclude the possibility that IL-17RC deficiency might negatively affect the development of CD8^+^ T cells in mice, we quantified CD8^+^ T cells as well as neutrophils, monocytes, B cells, and CD4^+^ T cells in spleen samples collected from *Il17rc*^*-/-*^ and WT mice and analyzed by flow cytometry. The results showed no difference in any of these cell populations in the spleen and blood from *Il17rc*^*-/-*^ and WT mice (S3 Fig), further confirming that the decrease of CD8^+^ T cell infiltration in the brain of *Il17rc*^*-/-*^ mice are due to the deficiency of the signaling of IL-17A through IL-17RC.

Our previous study has demonstrated that IL-17A promotes cytotoxicity of CD8^+^ T cells in combating WNV infection in mice [50]; therefore, we examined the expression of cytotoxicity gene mediators, such as perforin and granzyme B, in CD8^+^ T cells from *Il17rc*^*-/-*^ mice by flow cytometry. The results indicated no changes in the cell percentages and the MFI of the granzyme B expressing CD8^+^ T cells in *Il17rc*^*-/-*^ mice compared to WT mice (S4 Fig). However, a significant decrease in the MFI, but not the cell percentages, of the expression of perforin (Fig 3G and 3H) in CD8^+^ T cells was observed in *Il17rc*^*-/-*^ mice, suggesting that CD8^+^ T cells from *Il17rc*^*-/-*^ mice may have defects in cytotoxic effector function. To confirm this phenotype in the brain, we isolated CD8^+^ T cells from the brain-infiltrated leukocytes from *Il17rc*^*-/-*^ and WT mice infected with WNV. These CD8^+^ T cells (effector cells) were co-cultured with either WNV-E ectodomain expressing MC57GL cells (target cells) or control cells containing the empty vectors for 8 hours (h). The cytotoxicity of the effector CD8^+^ T cells against WNV-E-expressing target cells was determined by measuring the level of released lactate dehydrogenase (LDH) in the cell culture medium. The results showed that the cytotoxicity of the CD8^+^ T cells from *Il17rc*^*-/-*^ mice was significantly lower compared to that of WT mice (Fig 3I), suggesting that the CD8^+^ T cells of *Il17rc*^*-/-*^ mice are less cytotoxic in WNV-infected cells in the brain. To test whether this phenotype is not specific to brain-infiltrated CD8^+^ T cells, we also quantified the cell percentages and MFI of the perforin expression in splenic CD8^+^ T cells from WNV-infected WT and *Il17rc*^*-/-*^ mice. The results indicate that both splenic and brain-infiltrated CD8^+^ T cells of *Il17rc*^*-/-*^ mice produce less perforin compared to their counterparts from WT mice (Fig 3J and 3K). Collectively, these results indicate that IL-17A signaling through its receptor, IL-17RC, promotes the infiltration and cytotoxicity of CD8^+^ T cells in the brain.

### IL-17A-IL-17RA signaling increases the cytotoxicity of WNV-specific CD8^+^ T cells

**Fig 4:**
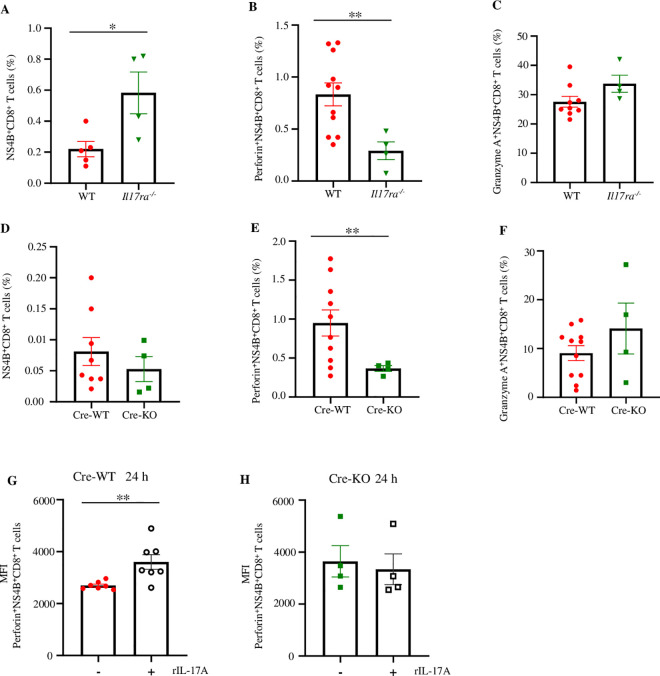
The effects of IL-17A-IL-17RA signaling on the cytotoxicity of WNV-specific CD8^+^ T cells. (A-F) Eight to nine weeks old *Il17ra*^*-/-*^ (n = 4), WT (n = 4 to 11), cre-WT (n = 8), and cre-KO mice (n = 4) were infected with 20 PFU of WNV via f.p. inoculation. Spleens were collected on D8 p.i. Splenocytes were stimulated with 0.01 MOI of WNV *in vitro* for 24 h with the addition of Brefeldin-A solution for the last 6 h of stimulation. Splenocytes were then characterized by flow cytometry after probing with WNV NS4B-tetramer, and antibody against CD8 followed by re-probing intracellularly with antibodies against perforin, and granzyme A. (A) The percentages of NS4B^+^CD8^+^ T cells of *Il17ra*^*-/-*^ and WT mice; (B) the expression of perforin^+^NS4B^+^CD8^+^ T cells; and (C) granzyme A^+^NS4B^+^CD8^+^ T cells from *Il17ra*^*-/-*^, and WT mice. (D) The percentages of NS4B^+^CD8^+^ T cells; (E) the expression of perforin^+^NS4B^+^CD8^+^ T cells; and (F) granzyme A^+^NS4B^+^CD8^+^ T cells from cre-KO, and cre-WT mice. (G-H) Eight to nine weeks old cre-WT (n = 7), and cre-KO mice (n = 4) were infected with 20 PFU of WNV via f.p. inoculation, and splenocytes were collected on D8 p.i. then treated with either rIL-17A (100 ng/ml) or RPMI (control) for 24 h and characterization by flow cytometry. (G) MFI of perforin^+^NS4B^+^CD8^+^ T cells in cre-WT mice. (H) MFI of perforin^+^NS4B^+^CD8^+^ T cells in cre-KO mice. Data were analyzed by two-tailed student’s *t*-tests (A-G), and Mann Whitney U tests (H) and presented as mean ± s.e.m. with *, and ** denoting *p* <0.05, and *p* <0.01, respectively.

In our previous study, we found that an *ex vivo* supplement of mouse rIL-17A augmented the gene expression of perforin and granzymes in the splenic CD8^+^ T cells isolated from WNV-infected mice deficient in IL-17A (*Il17a*^*-/-*^) and WT mice [50]. In the current study, we further investigated if IL-17A signaling via its receptors promotes cytotoxicity of WNV-specific CD8^+^ T cells from WNV-infected IL-17RA deficient (*Il17ra*^*-/-*^) mice. The mice were infected with 20 PFU of WNV via f.p. inoculation, and the spleens were collected on D8 p.i. We used a lower dose to infect the animals so that the animals remained alive for more days, which allowed us to collect the spleen at D8 p.i. when the WNV-specific CD8^+^ T cells become mature [50]. Splenocytes were stimulated with WNV *ex vivo,* and WNV-specific CD8^+^ T cells were identified using WNV-NS4B tetramer [50]. Perforin and granzyme A production in WNV-specific CD8^+^ T cells (NS4B^+^CD8^+^) was quantified with flow cytometry. There was a significant increase in the percentage of NS4B^+^CD8^+^ T cells in *Il17ra*^*-/-*^ mice (Fig 4A); however, the production perforin was lower in the NS4B^+^CD8^+^ T cells from *Il17ra*^*-/-*^ mice than in the WT controls (Fig 4B). There was no change in the level of granzyme A (Fig 4C). To confirm the phenotype, we infected *Il17ra* conditional knockout mice, which lack *Il17ra* expression on CD8^+^ cells (cre-KO mice) and their WT littermates’ control (cre-WT) with WNV as above. The flow cytometric results showed no changes in the splenic NS4B^+^CD8^+^ T cell numbers (Fig 4D) or granzyme A expression levels (Fig 4F), while a significant decrease was noted in perforin expression levels in the NS4B^+^CD8^+^ T cells from the cre-KO mice compared to the cre-WT controls (Fig 4E). These results suggest that the IL-17A-IL-17RA signaling axis is essential to the cytotoxicity of WNV-specific CD8^+^ T cells. To determine if a supplement of rIL-17A could promote the cytotoxicity of WNV-specific CD8^+^ T cells, we treated the splenocytes collected from WNV-infected cre-WT and cre-KO mice *ex vivo* and measured the expression of perforin in these cells by flow cytometry. Results showed that rIL-17A treatment increased the MFI of perforin^+^NS4B^+^CD8^+^ T cells from the WNV-infected cre-WT mice but not the cells from WNV-infected cre-KO mice (Fig 4G and 4H). Collectively, these results suggest that IL-17A signaling via the IL-17A-IL-17RA signaling axis can increase the cytotoxicity of WNV-specific CD8^+^ T cells both *in vivo* and *ex vivo*.

### IL-17A-IL-17RC signaling increases the cytotoxicity of WNV-specific CD8^+^ T cells

**Fig 5:**
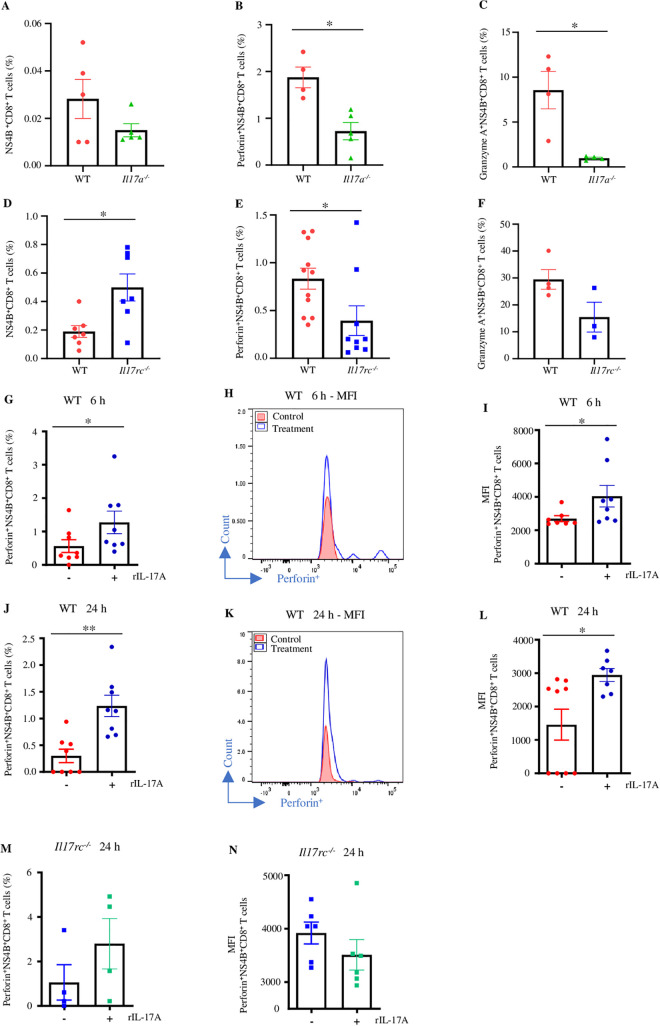
The effects of IL-17A signaling on the cytotoxicity of WNV-specific CD8^+^ T cells. Eight to nine weeks old *Il17a*^*-/-*^ (n = 5), *Il17rc*^*-/-*^ (n = 3 to 8), and WT (n = 4 to 11) mice were infected with 20 PFU of WNV via f.p. inoculation. Spleens were collected on D8 p.i., and splenocytes were stimulated with 0.01 MOI of WNV *in vitro* for 24 h, with the addition of Brefeldin-A solution for the last 6 h of stimulation. Splenocytes were then characterized by flow cytometry after probing with WNV NS4B-tetramer and antibody against CD8 followed by re-probing intracellularly with antibodies against perforin and granzyme A. (A) The percentages of NS4B^+^CD8^+^ T cells of *Il17a*^*-/-*^ and WT mice; (B) the expression of perforin^+^; and (C) granzyme A^+^NS4B^+^CD8^+^ T cells from *Il17a*^*-/-*^ and WT mice. (D) The percentages of NS4B^+^CD8^+^ T cells; (E) the expression of perforin^+^; and (F) granzyme A^+^NS4B^+^CD8^+^ T cells from *Il17rc*^*-/-*^ and WT mice. Eight to nine weeks old WT (n = 8) and *Il17rc*^*-/-*^ mice (n = 4 to 6) were infected with 20 PFU of WNV via f.p. inoculation, and splenocytes were collected on D8 p.i. then treated with either rIL-17A (100 ng/ml) or RPMI (control) for 6 or 24 h, and characterization by flow cytometry. (G) Perforin^+^NS4B^+^CD8^+^ T cell percentages; (H-I) histograms and values of MFI of perforin expression in 6 h treatment in WT mice. (J) Perforin^+^NS4B^+^CD8^+^ T cell percentages; (K-L) histograms and values of MFI of perforin expression in 24 h treatment in WT mice. (M) Perforin^+^NS4B^+^CD8^+^ T cell percentages; and (N) MFI of perforin expression in 24 h treatment in *Il17rc*^*-/-*^ mice. Data were analyzed by Mann Whitney U tests (A-C, F, and M), and two-tailed student’s *t*-tests (D-E, G, I-J, L, and N) and presented as mean ± s.e.m. with *, and ** denoting *p* <0.05, and *p* <0.01, respectively. The data represent at least two independent experiments.

Both IL-17RA and IL-17RC receptors are required for initiating IL-17A signaling, while IL-17RC is the IL-17A-specific receptor. Hence, we investigated if IL-17A signaling via IL-17RC promotes cytotoxicity of WNV-specific CD8^+^ T cells utilizing *Il17a*^*-/-*^ and *Il17rc*^*-/-*^ mice, which are deficient in IL-17A ligand and the receptor C, respectively. Eight to nine weeks old *Il17a*^*-/-*^, *Il17rc*^*-/-*^, and WT mice were infected with 20 PFU of WNV via f.p. inoculation, and the spleens were collected on D8 p.i. Splenocytes were stimulated with 0.01 MOI of WNV *ex vivo,* and WNV-specific CD8^+^ T cells were identified using WNV-NS4B tetramer [50]. Perforin and granzyme A production in WNV-specific CD8^+^ T cells (NS4B^+^CD8^+^) was quantified with flow cytometry. There was no significant change in the percentage of NS4B^+^CD8^+^ T cells (Fig 5A); however, the production of both perforin and granzyme A was dramatically reduced in the NS4B^+^CD8^+^ T cells from *Il17a*^*-/-*^ mice than in the WT controls (Fig 5B and 5C). Although *Il17rc*^*-/-*^ mice exhibited a higher percentage of NS4B^+^CD8^+^ T cells in the spleen (Fig 5D), the production of perforin was lower in these cells compared to WT controls (Fig 5E), and a similar trend was also detected for granzyme A, but it was not statistically significant (Fig 5F). To determine if a supplement of rIL-17A could also promote the cytotoxicity of WNV-specific CD8^+^ T cells, we treated the splenocytes collected from WNV-infected WT and *Il17rc*^*-/-*^ mice *ex vivo* and measured the expression of perforin in these cells by flow cytometry. Consistent with our previous findings, rIL-17A treatment enhanced the percentage of perforin^+^NS4B^+^CD8^+^ T cell populations from the WNV-infected WT mice at both 6 h and 24 h time points (Fig 5G and 5J). In addition, there was also an increase in the level of MFI in the WT NS4B^+^CD8^+^ T cell populations after rIL-17A treatment (Fig 5H, 5I, 5K, and 5L). In contrast, no change of perforin expression was detected in these cells isolated from WNV-infected *Il17rc*^*-/-*^ mice after rIL-17A treatment for 24 h (Fig 5M and 5N). These results suggest that the *ex vivo* treatment of IL-17A can increase the cytotoxicity of WNV-specific CD8^+^ T cells from WT mice but not from *Il17rc*^*-/-*^ mice, reinforcing the notion that IL-17A signals through IL-17RC to promote the cytotoxicity of CD8^+^ T cells during WNV infection.

### IL-17A signaling activates PI3K-mTOR signaling in CD8^+^ T cells

**Fig 6:**
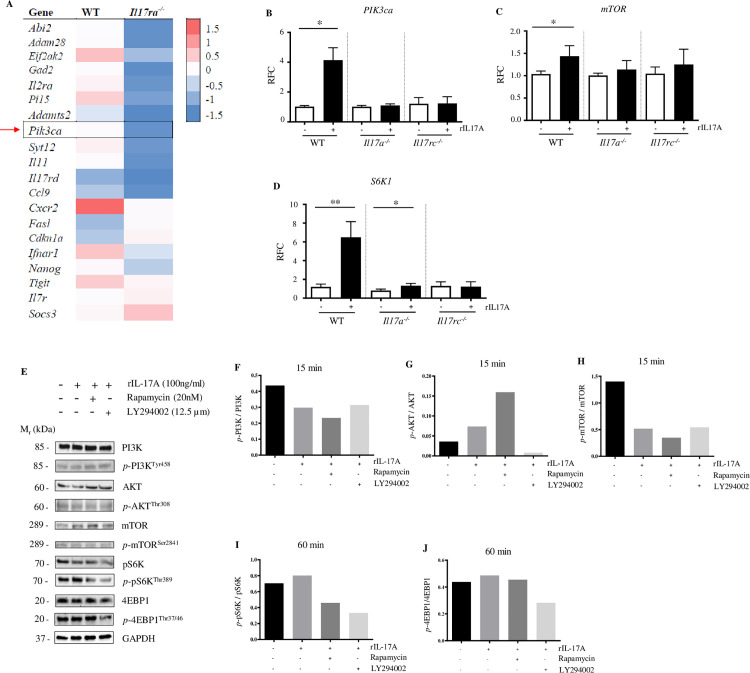
IL-17A enhances PI3K-mTOR signaling in CD8^+^ T cells. (A) Eight weeks old *Il17ra*^*-/-*^, and WT (C57BL/6J) mice (n = 3) were infected with 100 PFU of WNV via f.p. inoculation, and the spleens were collected on D8 p.i., followed by purification of CD8^+^ T cells. The transcription profile was analyzed by RNA sequencing and the representative data were shown as a heatmap. (B-D) Eight to nine weeks old *Il17a*^*-/-*^ (n = 5), *Il17rc*^*-/-*^ (n = 4), and WT (n = 8) mice were infected with 20 PFU of WNV via f.p. inoculation, and the spleens were collected on D8 p.i. CD8^+^ T cells were isolated from the splenocytes, cultured *ex vivo* with mouse rIL-17A (50 ng/ml) or RPMI (control) for 24 h and measured the gene expression of *PIK3ca* (B), *mTOR* (C), and *S6K1* (D) by RT-qPCR, expressed as the relative fold changes (RFC). Data were analyzed by two-tailed student’s *t-*tests (B-D) and presented as mean ± s.e.m. with *, and ** denoting *p* <0.05, and *p* <0.01, respectively. (E-J) Eight to nine weeks-old WT mice were infected with 20 PFU of WNV via f.p. inoculation, and the spleens were collected on D8 p.i. CD8^+^ T cells were isolated from the splenocytes, stimulated *ex vivo* with mouse rIL-17A (100 ng/ml) or RPMI (control), and co-treated with either rapamycin (20 nM), LY294002 (12.5 µM) for 15- or 60- minutes. Cell lysates were resolved with SDS-PAGE. The protein expressions of PI3K, *p*-PI3K^Tyr458^, AKT, *p*-AKT^Thr308^, mTOR, *p*-mTOR^Ser2481^, pS6K, *p*-pS6K^Thr389^, 4EBP1, and p-4EBP1^Thr37/46^ were analyzed by immunoblot. GAPDH was used as the loading control. (E) The protein bands of PI3K, *p*-PI3K^Tyr458^, AKT, *p*-AKT^Thr308^, mTOR, and *p*-mTOR^Ser2481^ were from the 15-min treatment, and the remaining bands were from the 60-min treatment. (F-J) Densitometric quantification of the phosphorylated protein normalized to corresponding total protein and analyzed by Image Lab software.

To understand the mechanism by which IL-17A signaling promotes cytotoxicity of CD8^+^ T cells, we analyzed the profiles of transcription in splenic CD8^+^ T cells collected from WNV-infected WT and *Il17ra*^*-/-*^ mice by RNA sequencing. IL-17RA is widely expressed in hematopoietic tissue, epithelial, endothelial, fibroblast cells, and lymphocytes, including CD8^+^ T cells [61]. The RNA sequencing data [62] showed that the PI3K-mTOR signaling may be activated by IL-17A signaling (Fig 6A). To verify if IL-17A signaling activates the PI3K-mTOR pathway, we treated CD8^+^ T cells isolated from splenocytes of WNV-infected *Il17a*^*-/-*^, *Il17rc*^*-/-*^, and WT mice with rIL-17A and measured the gene expression of *PIK3ca*, *mTOR*, and *S6K1* by RT-qPCR. The results indicate that rIL-17A treatment increases the expression of *PIK3ca*, *mTOR*, and *S6K1* in CD8^+^ T cells from WT mice but not from *Il17rc*^*-/-*^ mice (Fig 6B-D). In addition, treatment with rIL-17A also increased the expression of *S6K1* in CD8^+^ T cells from *Il17a*^*-/-*^ mice, albeit the expression of *PIK3ca* and *mTOR* showed a slight trend of increase after the treatment, but the change was not statistically significant (Fig 6B-D). Immunoblots were conducted to confirm that IL-17A signaling can activate the PI3K-mTOR pathway at the protein level. The splenic CD8^+^ T cells isolated from WNV-infected WT mice were treated with rIL-17A and simultaneously co-treated with either PI3K inhibitor (LY294002) [63] or mTOR inhibitor (Rapamycin) [64], or RPMI (control) *ex vivo.* Data showed that rIL-17A treatment slightly increases the production of phosphorated AKT, p70S6K (pS6K), and 4EBP1 at 15- or 60-minute (min) time points (Fig 6E, 6G, and 6I-J). Moreover, the presence of the inhibitors of PI3K and mTOR decreases the phosphorylation of pS6K, and 4EBP1, but not PI3K and mTOR (Fig 6E-J).

**Fig 7:**
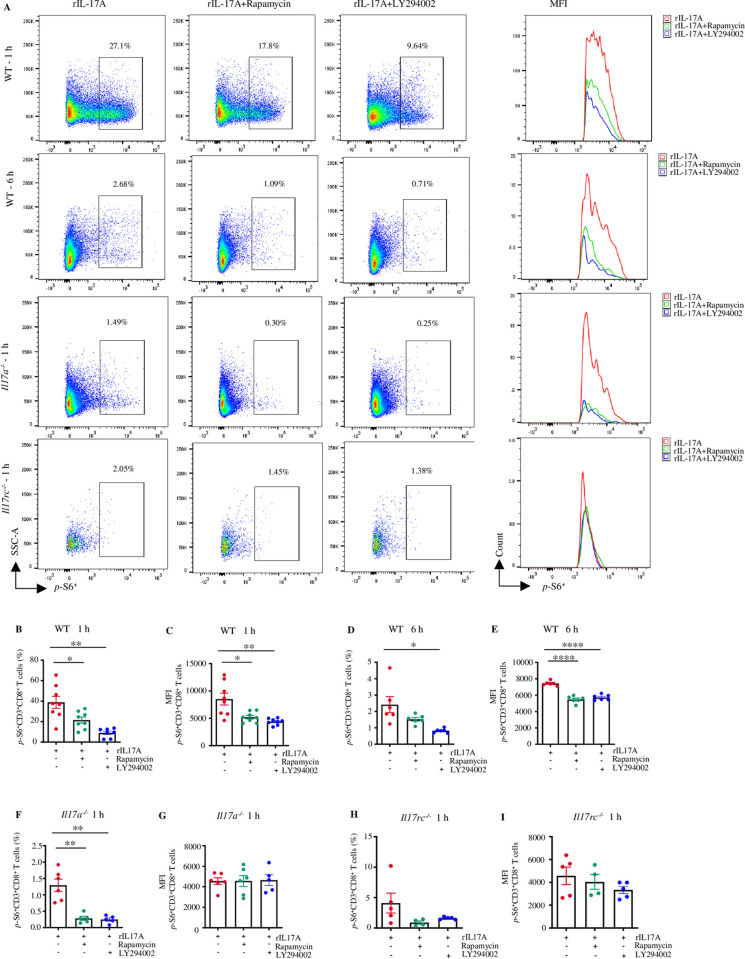
IL-17A activates PI3K-mTOR signaling at the substrate level in CD8^+^ T cells. (A-E) Splenocytes from WNV-infected WT mice (n=8) were stimulated with mouse rIL-17A (100 ng/ml) and treated with either rapamycin (20 nM), LY294002 (12.5 µM), or RPMI (control) for 1 or 6 h. (A) The representative pseudocolor plots and MFI of *p-*S6 of *p*-S6^+^CD3^+^CD8^+^ T cells by flow cytometry. (B-E) The flow cytometry results of *p-*S6 expression in *p*-S6^+^CD3^+^CD8^+^ T cells post 1 h or 6 h of treatment. (F-I) Splenocytes from WNV-infected *Il17a*^*-/-*^ (n=6) and *Il17rc*^*-/-*^ mice (n=5) were stimulated with mouse rIL-17A (100 ng/ml) and treated with either rapamycin (20 nM), LY294002 (12.5 µM), or RPMI (control) for 1 h. (F) Percentages of *p*-S6^+^CD3^+^CD8^+^ T cells and (G) MFI of *p*-S6^+^CD3^+^CD8^+^ T cells in *Il17a*^*-/-*^ mice. (H) Percentages *p*-S6^+^CD3^+^CD8^+^ T cells and (I) MFI of *p*-S6^+^CD3^+^CD8^+^ T cells in *Il17rc*^*-/-*^ mice. Data were analyzed by two-tailed student’s *t-*tests (B-G), and Mann Whitney U tests (H-I) and presented as mean ± s.e.m. with *, **, and **** denoting *p* <0.05, *p* <0.01, and *p* <0.0001, respectively. The data represent at least two independent experiments.

Once the PI3K-mTOR pathway gets activated, there is phosphorylation of effector molecules such as pS6K, and 4EBP1. The phosphorylated pS6K (*p*-S6K) activates the downstream signal transduction by phosphorylating its substrate, the ribosomal S6 protein, thereby increasing the production of phosphorylated S6 (*p*-S6) [65]. To further confirm that IL-17A activates the PI3K-mTOR pathway, we assessed the level of *p*-S6 in splenic CD8^+^ T cells from WNV-infected WT, *Il17a*^*-/-*^, and *Il17rc*^*-/-*^ mice by flow cytometry. The splenocytes were treated with rIL-17A and simultaneously co-treated with either PI3K inhibitor (LY294002) or mTOR inhibitor (Rapamycin), or RPMI (control) *ex vivo* for 1 or 6 h*.* The results demonstrated that adding PI3K and mTOR inhibitors in the presence of rIL-17A reduced the expression of *p*-S6 in CD8^+^ T cells from WT, and *Il17a*^*-/-*^ but not from *Il17rc*^*-/-*^ mice (Fig 7A-I). The flow cytometry results confirm that IL-17A signaling activates the PI3K-mTOR pathway at the substate level. Thus, these findings collectively suggest that IL-17A activates PI3K-mTOR signaling in CD8^+^ T cells during WNV infection.

### IL-17A signaling stimulates metabolism in CD8^+^ T cells during WNV infection

**Fig 8:**
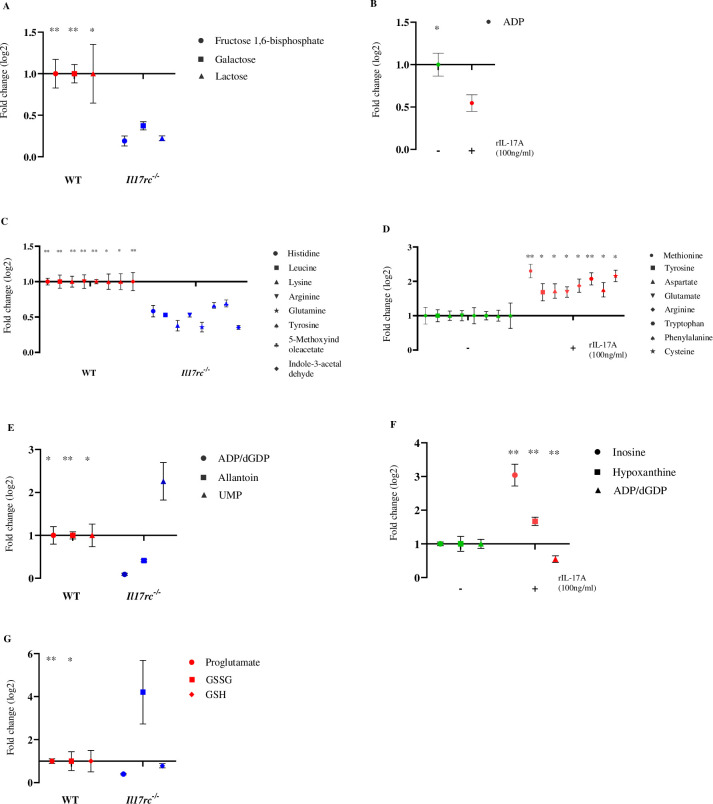
IL-17A signaling promotes metabolism of CD8^+^ T cells. Eight to nine weeks old *Il17rc*^*-/-*^ (n = 3) and WT (n = 3) mice were infected with 20 PFU of WNV via f.p. inoculation, and the spleens were collected on D8 p.i. Levels of metabolites of glucose (A), amino acid (C), nucleotide (E), and redox homeostasis (G) in the purified CD8^+^ T cells were measured by mass spectrometry. The CD8^+^ T cells from the WT mice were treated with mouse rIL-17A (100 ng/ml) or RPMI (control) for 24 h, and the levels of metabolites of glucose (B), amino acid (D), and nucleotide metabolism (F) were measured by mass spectrometry. Data were analyzed by one-tailed student’s *t*-tests and presented as mean ± s.e.m. with * and ** denoting *p* <0.05, and *p* <0.01, respectively.

It has been reported that the activation of the PI3K-mTOR signaling pathway leads to increased metabolism in CD8^+^ T cells to cope with the higher energy demand in the inflammatory microenvironment and promotes CD8^+^ T cell effector function [61,66–68]. Therefore, we evaluated whether IL-17A signaling promotes metabolism in CD8^+^ T cells during WNV infection. Global profiling of glucose, amino acids, and nucleotides metabolism was performed on CD8^+^ T cells purified from WNV-infected WT and *Il17rc*^*-/-*^ mice with or without rIL-17A treatment by liquid chromatography-mass spectrometry (LC-MS/MS). The metabolomic results showed that the expression levels of fructose 1,6 bisphosphate (FBP), galactose, and lactose in CD8^+^ T cells from *Il17rc*^*-/-*^ mice were lower than those in WT mice (Fig 8A). FBP is generated from phosphorylation of fructose-6-phosphate (F6P) by phosphofructokinase-1 (PFK-1). FBP directly increases aerobic glycolysis by stimulating pyruvate kinase, a key rate-limiting glycolytic enzyme [69]. During FBP production, a fraction of F6P bypasses glycolysis to be converted into fructose 2,6-bisphosphate (F2,6-BP) by PFK-2. F2,6-BP acts as an allosteric activator of PFK-1, increasing aerobic glycolysis [69,70]. Consequently, with the help of PFK-1, FBP indirectly increases glycolytic flux to support higher proliferation. Thus, the reduced level of FBP suggests a decrease in aerobic glycolysis within CD8^+^ T cells of *Il17rc*^*-/-*^ mice, implying that IL-17A signaling increases aerobic glycolysis.

During anaerobic glycolysis, a newly formed pyruvate undergoes reduction to lactate by LDH in the cytosol, along with the generation of two adenosine triphosphate (ATP) molecules per glucose. Therefore, a lower lactate level indicates a decline in anaerobic glycolysis and gluconeogenesis in CD8^+^ T cells of *Il17rc*^*-/-*^ mice (Fig 8A). In addition, decreased galactose, a substrate for lactose, also results in a reduction in anaerobic glycolysis. Thus, the results suggest that IL-17A-IL-17RC signaling is required for both aerobic and anaerobic glycolysis in CD8^+^ T cells. This hypothesis is supported by the *ex vivo* rIL-17A treatment results, showing that the rIL-17A treatment decreases the production of adenosine diphosphate (ADP), an inhibitor of glycolytic enzymes [69], which in turn increases aerobic glycolysis in CD8^+^ T cells of WT mice (Fig 8B).

Activated CD8^+^ T cells also require an augmented influx of various amino acids to support the effector functions. Next, we examined if IL-17A signaling alters the amino acid metabolism in CD8^+^ T cells from WNV-infected *Il17rc*^*-/-*^ and WT mice. Compared with those from WT mice, the levels of histidine, leucine, lysine, arginine (Arg), glutamine, and tyrosine (Tyr) in CD8^+^ T cells from *Il17rc*^*-/-*^ mice are significantly decreased (Fig 8C). The increase of amino acid metabolism augmented by IL-17A signaling was also confirmed by the *ex vivo* rIL-17A treatment data showing significant increases in methionine, Tyr, aspartate, glutamate, Arg, tryptophan, phenylalanine, and cysteine in CD8^+^ T cells from WT mice (Fig 8D). Thus, these data suggest that IL-17A signaling increases amino acid metabolism.

To evaluate nucleotide metabolism in CD8^+^ T cells, we analyzed the metabolites related to purine and pyrimidine metabolism. The data showed a significant reduction of the levels of adenosine diphosphate/deoxyguanosine diphosphate (ADP/dGDP), and allantoin in CD8^+^ T cells from *Il17rc*^*-/-*^ mice compared to those from WT mice (Fig 8E). ADP/dGDP decreases both *de novo* and salvage purine synthesis via feedback inhibition of 5-phosphoribosyl-α-1-pyrophosphate (PRPP), the rate-limiting substrate for both pathways [71]. Allantoin is a metabolic intermediate of purine catabolism produced from the oxidation of uric acid [72]. Thus, decreases in ADP/dGDP and allantoin in CD8^+^ T cells from *Il17rc*^*-/-*^ mice suggest more purine synthesis and less purine breakdown, respectively. The data from pyrimidine metabolism showed a significant increase in uridine 5′ monophosphate (UMP) level in CD8^+^ T cells of *Il17rc*^*-/-*^ mice compared to WT mice (Fig 8E). High levels of UMP decrease *de novo* pyrimidine biosynthesis by feedback inhibition of carbamoyl phosphate synthase (CPS)- II, the primary regulatory enzyme of pyrimidine synthesis [73,74]. Therefore, decreased *de novo* pyrimidine synthesis in *Il17rc*^*-/-*^ mice implies that IL-17A signaling may increase pyrimidine metabolism. We checked if *ex vivo* rIL-17A treatment can affect nucleotide metabolism to confirm the results. Data on the purine metabolism showed increased inosine and hypoxanthine levels in the rIL-17A treatment group in WT mice (Fig 8F). Hypoxanthine, derived from inosine, is an important metabolic intermediate, required for salvage purine synthesis [71]. Interestingly, rIL-17A treatment also significantly inhibits the production of ADP/dGDP (Fig 8F), which is the feedback inhibitor of both *de novo* and salvage purine synthesis [75]. These results suggest that IL-17A signaling enhances the nucleotide metabolism in CD8^+^ T cells during WNV infection.

During T-cell activation, higher metabolic demand initiates an excess production of reactive oxygen species (ROS), resulting in the toxic accumulation of ROS and triggering oxidative stress in the cells [76]. In responding to oxidative stress, antioxidants, particularly reduced glutathione (GSH), are produced to neutralize ROS [76]. GSH neutralizes the ROS by continuously supplying reducing equivalents and undergoes conversion into oxidized glutathione (GSSG). Sustenance of the reduced state of other antioxidants is also maintained via GSH through a redox cycling reaction [77]. To assess the oxidative stress in the purified CD8^+^ T cells from WNV-infected *Il17rc*^*-/-*^ and WT mice, we measured the levels of GSH, GSSG, and pyroglutamate, a substrate for GSH synthesis. The results indicate a reduction in GSH levels and an increase in GSSG in *Il17rc*^*-/-*^ mice (Fig 8G). This suggests that oxidative stress is elevated in these mice, leading to an increase in ROS production. The ROS are neutralized by GSH but are simultaneously oxidized into GSSG. Consequently, there is a decrease in GSH levels and an increase in GSSG. Additionally, the lower pyroglutamate levels in *Il17rc*^*-/-*^ mice are associated with reduced GSH production, which further contributes to oxidative stress. Hence, these results collectively indicate that the CD8^+^ T cells from *Il17rc*^*-/-*^ mice exhibit a higher ratio of GSSG to GSH and lower pyroglutamate levels, suggesting increased oxidative stress in these cells compared to those from WT mice during WNV infection (Fig 8G). Therefore, it is reasonable to conclude that IL-17A signaling reduces oxidative stress by maintaining redox homeostasis and increasing glutathione metabolism in CD8^+^ T cells.

### IL-17A-mediated PI3K-mTOR signaling increases the cytotoxicity of CD8^+^ T cells

**Fig 9:**
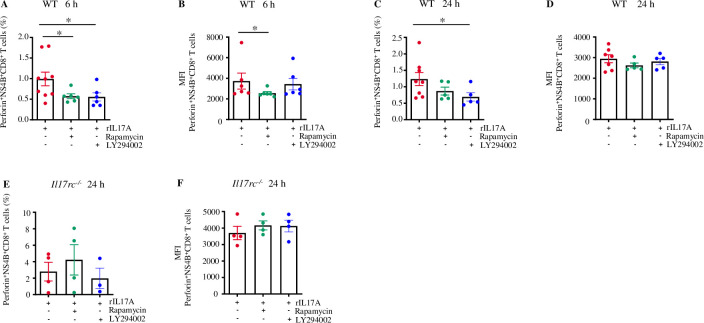
IL-17A signaling promotes perforin expression by activating the PI3K-mTOR pathway in WNV-specific CD8+ T cells. Eight to nine weeks old WT (n = 7 to 8) and *Il17rc*^*-/-*^ mice (n = 4) were infected with 20 PFU of WNV via f.p. inoculation, and the spleens were collected on D8 p.i. The collected splenocytes were treated with mouse rIL-17A (100 ng/ml) with or without rapamycin (20 nM) and LY294002 (12.5 µM) for 6 or 24 h and characterized by flow cytometry after probing with WNV NS4B tetramer and antibodies against CD8 and perforin. The perforin^+^NS4B^+^CD8^+^ T cell percentages (A), MFI (B) of 6 h treatment; the percentages (C), and MFI (D) of 24 h treatment in WT mice. The data represent at least two independent experiments. The percentages of perforin^+^NS4B^+^CD8^+^ T cells (E) and MFI (F) for 24 h treatment in *Il17rc*^*-/-*^ mice. Data were analyzed by two-tailed student’s *t*-tests and presented as mean ± s.e.m. with * denoting *p* <0.05.

Next, we evaluated the effect of IL-17A-mediated PI3K/mTOR signaling activation on the cytotoxicity of WNV-specific CD8^+^ T cells. The splenocytes were isolated from WNV-infected WT and *Il17rc*^*-/-*^ mice on D8 p.i. and treated with rIL-17A in the presence or absence of rapamycin and LY294002 for 6 or 24 h *ex vivo.* The production of perforin in the NS4B^+^CD8^+^ T cells was quantified by flow cytometry. The results showed a decrease in perforin production in the WT cells with the rapamycin treatment at 6 h (Fig 9A and 9B). A similar reduction trend was also detected at 24 h treatment, although the difference is not statistically significant (Fig 9C and 9D). The treatment of LY294002 decreased the percentage of perforin-expressing NS4B^+^CD8^+^ T cells compared to the rIL-17A treatment control group at both 6 and 24 h time points (Fig 9A and 9C). As expected, no changes in the perforin expression were observed in either rapamycin or LY294002 treatment groups in NS4B^+^CD8^+^ T cells from *Il17rc*^*-/-*^ mice (Fig 9E and 9F), indicating that IL-17RC was required for the activation of IL-17A-mediated PI3K-mTOR signaling. Hence, these results collectively suggest that IL-17A signaling-mediated PI3K-mTOR pathway can promote cytotoxicity in CD8^+^ T cells during WNV infection.

## Discussion

CD8^+^ T cells recognize peptide-class I major histocompatibility complexes (MHC) and play a key role in both clearing acute viral infection and generating long-lived memory cells primed to provide a rapid response to repeated infections [78]. CD8^+^ T cells control viral infections by triggering apoptosis of infected cells via perforin or Fas ligand-dependent pathways or producing antiviral cytokines (e.g., IFN-γ and TNF-α) [78]. Infection with WNV can lead to life-threatening encephalitis and meningitis, and CD8^+^ T cells are essential in clearing WNV infection from the CNS of humans and experimental mice. Mice deficient in CD8^+^ T cells or class I MHC antigens had higher viral burdens in the CNS and increased mortality rates. The absence of CD8^+^ T cells did not affect the kinetics or magnitude of viremia nor the production of anti-WNV antibodies [79]. In addition, WNV infection of mice deficient in perforin, Fas ligand, and TNF-related apoptosis-inducing ligand (TRAIL) resulted in an increased viral burden in the CNS and mortality following infection [80–82]. In contrast, anti-viral antibodies and B cells are essential in protecting against WNV infection by controlling viremia and viral loads in the peripheral organs, thus limiting viral dissemination into the CNS. Prophylactic and therapeutic transfer of human IgG or mouse serum delayed or protected mice from WNV infection. However, antibodies alone cannot eliminate viral reservoirs in host tissues, further suggesting CD8^+^ T cell-mediated cellular immune responses are required for WNV clearance [79,83]. Although some evidence suggests that CD8^+^ T cells might contribute to neuronal damage in several inflammatory, neurodegenerative, and paraneoplastic neurological disorders [84], in the case of WNV infection, CD8^+^ T lymphocytes play a crucial protective role in eliminating virus-infected cells [79] by inducing robust anti-viral adaptive immunity [85]. Considering the risk vs benefit of the cytotoxic potential of CD8^+^ T cells in the CNS, it is crucial to balance their ability to target and eliminate virus-infected cells with unintended damage to the healthy neuronal tissue. Careful modulation and control of CD8^+^ T cells are important to optimize their effector function. However, the regulation of their effector functions remains poorly understood. Through investigating WNV pathogenesis, we have uncovered a novel function of IL-17A signaling in promoting the cytotoxicity of CD8^+^ T cells. Importantly, a single injection of 2.5 μg of rIL-17A into WT mice on day 6 after WNV infection significantly increased expression of the cytotoxic mediator genes in CD8^+^ T cells and profoundly reduced viral burden in the brain and increased animal survival, suggesting a great therapeutic potential of IL-17A against WNV infection in the CNS [50]. Therefore, understanding how IL-17A signaling promotes the cytotoxicity of CD8^+^ T cells paves the way for applying IL-17A as a potential therapeutic agent to treat WNV infection in the CNS.

IL-17A induces signaling transduction through a heterodimer of IL-17RA and IL-17RC complex. While IL-17RA participates in the signaling pathways of other members of the IL-17 family, such as IL-17B, C, and E, IL-17RC is specific to the signaling of IL-17A, IL-17F, and IL-17A/F. IL-17F has been reported to have similar biological functions as IL-17A [86]. Thus, we utilized *Il17rc*^*-/-*^ mice to study how IL-17A signaling regulates the cytotoxicity of CD8^+^ T cells against WNV infection. After WNV infection, *Il17rc*^*-/-*^ mice bear a higher viral burden in the brain than the WT mice, although the viral load in the blood, liver, and spleen samples showed no apparent difference. In addition, the TUNEL assay results showed more apoptotic bodies in the brains of *Il17rc*^*-/-*^ mice. These findings suggest that IL-17A signaling is crucial to protect mice against WNV infection in the brain. During WNV neuroinvasion, monocytes, neutrophils, NK cells, DC cells, and effector CD4^+^ and CD8^+^ T cells were found to infiltrate the brain to respond to the infection [87]. Of these, monocytes have been reported as the most predominant immune cells infiltrated into the brains of WNV-infected mice [87,88]. Neutrophils exhibit a dual role in the pathogenesis of WNV, serving as viral reservoirs, facilitating the early viral spread to the CNS, and subsequently inhibiting viral replication [89]. Our flow cytometry results showed that the numbers of infiltrated CD8^+^ T cells were reduced compared to the WT control mice, suggesting that the higher susceptibility of *Il17rc*^*-/-*^ mice to WNV infection may be due to the lower magnitude of the functions of CD8^+^ T cells. The flow cytometry results also excluded the possibility that the absence of IL-17RC might negatively affect CD8^+^ T cell development, thus further supporting the notion that IL-17A signaling is essential to the effector functions of CD8^+^ T cells. Effector CD8^+^ T cells destroy target cells by releasing several cytotoxic mediators, such as perforin and granzymes. While perforin binds to the target cell membrane to form pores, damaging the integrity of the target cell membrane [90], granzymes activate apoptosis pathways after entering the target cells through the perforin pores [78]. In addition, effector CD8^+^ T cells can initiate the Fas-FasL interaction, triggering the caspase cascade and inducing apoptosis of the target cells [91,92]. The flow cytometry results showed that the expression of perforin in the brain-infiltrated CD8^+^ T cells from *Il17rc*^*-/-*^ mice was significantly lower than that of the WT mice, suggesting that IL-17A signaling is indispensable to maintaining the cytotoxicity of CD8^+^ T cells. A previous report indicated that the absence of CD8^+^ T cells in mice did not affect the kinetics or magnitude of WNV viremia but did increase the viral burden in the brain and mortality [79]. This aligns with our findings that *Il17rc*^*-/-*^ mice exhibited a higher viral burden in the brain compared to WT mice while maintaining similar viral loads in the blood and peripheral organs. One possible explanation is that WNV burdens in the blood and peripheral organs of mice typically peak between D3 and D4 p.i. before WNV-specific CD8^+^ T cells have matured and become functional [57–59].

It is worth noting that we used 100 PFU of infection via footpad inoculation to assess the susceptibility of *IL17rc*^*-/-*^ mice, measuring viral load, cytokine profiles, and immune cell brain infiltration as described in our previous publications [50,57,93]. This optimal dose was chosen because it results in approximately 50% mortality in WT C57BL/6 mice aged 7–8 weeks. Due to the variability among animals, even within the same group, some may exhibit sickness sooner and die, while others survive the infection. In this study, we collected brain and spleen samples on day 7 post-infection (p.i.), when WNV typically infects the brain, and all the animals in both groups are still alive, as death usually occurs between days 8 and 15 post-infection. Although some animals may start showing signs of sickness on day 7, most appear healthy. By collecting and analyzing samples from each surviving animal in both groups, we ensured unbiased sample collection and data presentation regardless of their health status. Any observed differences between WT and the knockout groups should reflect their immune responses to WNV infection. However, we cannot exclude the possibility that clinical heterogeneity within the same group may affect the measurements and data interpretation.

To further investigate whether IL-17A signaling promotes the cytotoxicity of WNV-specific CD8^+^ T cells via the IL-17A-IL-17RC axis, we evaluated the expression of perforin and granzyme A and the percentages of WNV NS4B tetramer and CD8 double-positive (NS4B^+^CD8^+^) cells from WNV-infected *Il17a*^*-/-*^ and *Il17rc*^*-/-*^ mice. The results of both mouse models suggest that IL-17A signaling promotes the cytotoxicity of WNV-specific CD8^+^ T cells. Interestingly, we noticed a significant increase in the percentage of NS4B^+^CD8^+^ T cells in *Il17rc*^*-/-*^ compared to WT mice. The higher number of NS4B^+^CD8^+^ T cells but lower cytotoxicity in *Il17rc*^*-/-*^ mice further implied that these WNV-specific CD8^+^ T cells were functionally defective. The *ex vivo* rIL-17A treatment experiment results confirmed that rIL-17A increased the perforin expression in the WNV-specific CD8^+^ T cells isolated from WT mice but not *Il17rc*^*-/-*^ mice. Thus, these findings further demonstrate the essential role of the IL-17A-IL-17RC axis on the cytotoxicity in WNV-specific CD8^+^ T cells. In addition, we did not notice any noticeable change in the expression of granzyme A, suggesting that the IL-17A signaling may be more effective in inducing perforin expression than granzyme A. Consistent with our findings on the roles of perforin, previous studies have demonstrated that CD8^+^ T cells require perforin to clear viral infections, including WNV from infected neurons [82,94,95]. Mice deficient in perforin resulted in a higher viral burden in the CNS and increased mortality after WNV infection [82]. Although perforin and granzyme A are key components of the cytotoxic machinery in CD8^+^ T cells, literature suggests their expression can be regulated independently under different conditions. For instance, IL-2 has been found to increase the expression of both perforin and granzyme A in CD8^+^ T cells, but the regulation of these molecules can occur independently [96]. Additionally, most CD8^+^ T cells in rectal tissue, including HIV (human immunodeficiency virus)-1-specific cells, fail to express perforin but granzyme A, indicating differential expression patterns of perforin and granzyme A [97]. The detailed molecular mechanisms of why IL-17A-IL17RC signaling may be more effective in inducing perforin expression than granzyme A are warranted for further investigation.

During a viral infection, effector T cells experience an increased influx of nutrients due to higher energy demands [66]. mTOR serves as a molecular sensor of metabolism that integrates signals associated with both immune activation and metabolic requirements [66]. Additionally, mTOR signaling via the mTORC1 complex not only induces differentiation of the effector over the memory lineage of CD8^+^ T cells but also prompts their cytotoxic effector function by inducing the expression of T-bet [98]. The CD28-mediated costimulatory signal required for activating CD8^+^ T cells during antigenic stimulation also serves as a classic signal for activating the PI3K-AKT-mTOR signaling pathway [61,66,67]. The engagement of TCR-MHC class I complex also activates PI3K in the activated CD8^+^ T cells [67]. Similar to the mTOR pathway, PI3K-AKT signaling also promotes the activation and differentiation of effector CD8^+^ T cells [67]. It has been reported that IL-17A via the IL-17RA axis activates the PI3K-mTOR pathway in mouse alveolar epithelial cells and promotes fibrosis [99]. Using a bleomycin-induced IL-17A activation model, Cong *et al.* also reported that IL-17A-IL-17RA signaling could activate the PI3K-AKT-mTOR signaling and increase pulmonary inflammation and fibrosis in mouse bronchial epithelial cells [61,66,67]. In the current study, our results suggest that IL-17A signaling upregulates the expression of *PI3K3ca*, *mTOR*, and *S6K1*, suggesting that IL-17A signaling may activate the PI3K-mTOR signaling pathway and increase the activity of *p*-pS6K. This notion is supported by evidence that rIL-17A treatment increases, while rapamycin and LY294002 inhibit the phosphorylation of pS6K in CD8^+^ T cells from WNV-infected WT mice in the immuno-blotting analysis. Moreover, IL-17A treatment also increases the phosphorylation of 4EBP1 at the protein level. Thus, IL-17A may affect the PI3K/mTOR signaling pathway via mediating phosphorylation of both downstream effectors, pS6K and 4EBP1.

Upon viral antigen activation, naïve CD8^+^ T cells undergo rapid differentiation into activated CD8^+^ T cells, subsequently engaging in swift proliferation to generate a pool of effector CD8^+^ T cells. This process requires higher energy and a continuous supply of different macromolecules as building blocks to generate robust antiviral responses. This increased energy demand is met by a radical shift in metabolism, transitioning from a resting catabolic state to a highly proliferative anabolic state [68]. While the naïve CD8^+^ T cells mainly rely on fatty acid oxidation and oxidative phosphorylation (OXPHOS), the short-lived effector CD8^+^ T cells differentiated due to any type of cellular insult, depend on both aerobic and anaerobic glycolysis [100], glutaminolysis, protein, and nucleotide synthesis [68]. It has been reported that PI3K-mTOR signaling acts as a metabolic switch for the differentiation and proliferation of CD8^+^ T cells to promote the effector functions [66]. However, the impact of IL-17A signaling on the metabolism of CD8^+^ T cells has not been studied. To address this question, we measured metabolites related to glucose, amino acids, nucleotides, and redox metabolism with LC-MS. Since fatty acid oxidation is primarily utilized by naïve CD8^+^ T cells for energy production [101], lipid metabolism is not included in the analysis. Data from the metabolomic study demonstrated that IL-17A signaling elevated both aerobic and anaerobic glycolysis in CD8^+^ T cells, which were further confirmed by the *ex vivo* rIL-17A treatment study on the cells from WNV-infected WT mice. T cells have been documented to metabolize glucose through both aerobic and anaerobic glycolysis during proliferation [100]. It has been reported that PI3K-AKT-mTORC1 signaling increases glycolysis in metabolically demanding conditions in actively proliferating immune cells [102,103]. Thus, our results suggest that IL-17A signaling activates the PI3K-mTOR pathway, leading to the increase of glycolysis in CD8^+^ T cells during WNV infection. Similarly, it also increases the metabolism of amino acids and nucleotides within CD8^+^ T cells during WNV infection. T-cell activation promotes the uptake of different amino acids, activating mTOR signaling, thereby promoting protein synthesis and metabolic reprogramming [104]. In addition to upholding structural integrity, amino acids are also required to participate in nucleotide metabolism, redox homeostasis, and epigenetic modifications of histones within T cells [104,105]. While purine and pyrimidine regulate the cell cycle differently, there is evidence suggesting that both nucleotides contribute to T cell proliferation and survival [106]. In highly proliferative T cells, mTOR, as a master regulator, promotes amino acid influx required for purinosomes formation and upregulates purine synthesis through the tetrahydrofolate cycle [106,107]. Similarly, activation of PI3K-mTOR signaling phosphorylates the trifunctional enzymes, i.e., CPS, aspartate carbamoyltransferase, and dihydroorotase, resulting in the initiation of pyrimidine synthesis [74]. Therefore, our results suggest that IL-17A signaling activates PI3K-mTOR signaling and subsequently increases the metabolism of glycolysis, amino acids, and nucleotides, enhancing the effector functions of CD8^+^ T cells.

Finally, we confirm if IL-17A signaling augments the cytotoxicity of CD8^+^ T cells via activating the PI3K-mTOR signaling pathway with their inhibitors, rapamycin and LY294002. The results demonstrated that IL-17A-mediated increase of the perforin expression was suppressed by both rapamycin and LY294002 in CD8^+^ T cells from WT but not in *Il17rc*^*-/-*^ mice. Thus, collectively, these results suggest that IL-17A signaling activates PI3K-mTOR-mediated metabolism and increases the cytotoxicity of CD8^+^ T cells for WNV clearance in the CNS. This study contributes by elucidating a novel mechanism of IL-17A signaling in WNV pathogenesis, adding valuable insights to the current understanding of the complex interplay between IL-17A and WNV infection.

## Methods

### Ethical statement and biosafety

The animal experiments in this study were reviewed and approved by the Institutional Animal Care and Use Committees (IACUC) at the University of Southern Mississippi (USM) under the IACUC protocols # 15101601 and 17110903. The experiments involving live WNV were performed by certified personnel in the Biosafety Level-2 (BSL-2, cell culture) and BSL-3 (animal) laboratories, following the biosafety protocols approved by the USM Institutional Biosafety Committee.

### Cells and viruses

Vero cells (ATCC CCL-81) were maintained in Dulbecco’s Modified Eagle’s Medium (DMEM, Life Technologies) supplemented with 1% Penicillin/Streptomycin (P/S, Gibco) and 10% Fetal Bovine Serum (FBS, Atlanta Biologicals). The splenic CD8^+^ T cells were maintained in RPMI 1640 medium (Gibco) supplemented with 1% P/S and 10% FBS. The cells were kept in an incubator at 37°C with 5% CO_2_ and a relative humidity of 95%. The WNV isolate CT2741 [108] was kindly provided by John F. Anderson at the Connecticut Agricultural Experiment Station, propagated in Vero cells, and quantified by a plaque-forming assay as previously described [109,110].

### Mice and animal studies

The breeding pair of C57BL/6J (WT) mice were purchased from the Jackson Laboratory (Bar Harbor, ME). The breeding pair of *Il17a*^*-/-*^ mice were kindly provided by Richard A. Flavell at the Yale University School of Medicine, and *Il17rc*^*-/-*^ mice by Dr. Zongbing You at Tulane University School of Medicine [55], and *Il17ra*^*-/-*^ mice by Dr. Sarah Gaffen at the University of Pittsburgh. All the knockout strains are in the C57BL/6J background. The breeding pairs and pups were kept in a clean room until they were ready to be used. The infection experiments were performed in an animal BSL-3 lab at USM. Seven to nine weeks old *Il17a*^*-/-*^, *Il17rc*^*-/-*^, and WT mice were subcutaneously injected on the ventral side of the right hind footpad with either 20 or 100 PFU of WNV in 50 µl of phosphate buffer saline (PBS) containing 5% gelatin after 30% isoflurane anesthesia [93]. In the survival experiments, the infected animals were monitored daily for 21 days for morbidity and mortality. Blood samples were collected from the retro-orbital sinus on D2 and 4 p.i. in 0.5M EDTA.

### Reverse transcription quantitative polymerase chain reaction (RT-qPCR)

The total RNA was extracted from cells and tissue samples using TRI Reagent (Molecular Research Center, Inc) and converted into the first-strand complementary DNA (cDNA) using the iSCRIPT cDNA synthesis kit (Bio-Rad). The RT-qPCR assays were performed in a CFX Connect Real-Time System (Bio-Rad) using iTaq Universal SYBR Green Supermix (Bio-Rad). The *WNV-E* RNA copies were measured by probe-based RT-qPCR, and data were normalized by mouse *β-actin* as a housekeeping gene [50,109]. Genes of various cytokines, CD8^+^ T cell cytotoxicity mediators, and PI3K-mTOR signaling mediators were quantified by SYBR Green-based RT-qPCR, and data were normalized by the ΔΔ*C*_*T*_ method using *β-actin* as the house-keeping gene to determine relative fold change (RFC) [50].

### Immunofluorescence assay (IFA)

Eight to nine weeks old *Il17rc*^*-/-*^ and WT mice were infected with 100 PFU of WNV via f.p. inoculation. Mice were anesthetized using 30% isoflurane on D7 p.i. and transcardially perfused with 30 ml of ice-cold PBS. The brains of the infected animals were then collected, fixed with 4% PFA, and cryoprotected with 20% followed by 30% sucrose for 24 h at 4°C. The brains were then mounted with OCT compound by snap freezing with liquid nitrogen and incubated overnight at -80°C. Brain sections were cut sagittally using a cryostat (Leica Biosystems) at a thickness of 10 µm, transferred to charged glass slides (Fisher Scientific), and air-dried overnight. The brain sections were blocked with 5% goat serum and 0.5% Triton X-100 in PBS for 1 h at 4°C, probed with rabbit anti-WNV-E polyclonal antibody (1:100, PA1–41073, Invitrogen) overnight at 4°C. The sections were washed 3 times with PBST for 10 min each. Then, the sections were stained with Alexa Fluor 568-conjugated goat anti-rabbit IgG (1:500, Invitrogen, A-11011) for 1 h at RT. The sections were washed as above and stained with DAPI (4´, 6-diamidino-2-phenylindole, Life Technologies) [111,112]. The images were acquired at 40x magnification using a Nikon AIR confocal microscope (Leica Biosystems).

### TUNEL assay

The TUNEL assay was conducted using a TACS 2 TdT-Fluor *In Situ* Apoptosis Detection Kit (R&D Systems). The brain samples were processed and sectioned into 10 µm thickness following the procedure mentioned in IFA. The cryosections are then rehydrated gradually with 100, 95, and 70% ethanol. After a PBS wash, the samples were treated with 50 µl Cytonin for 30 min at RT. The samples were then washed with labeling solution, followed by incubating with 50 µl of labeling mixture containing dNTP, TdT, manganese ion, and labeling buffer for 2 h at 37°C. The reactions were stopped with TdT stop buffer, washed twice, and stained with Strep-Fluorescein solution for 30 min in the dark at RT. Cell nuclei were stained with DAPI, and the images were acquired at 40x magnification using a Nikon AIR confocal microscope (Leica Biosystems).

### Flow cytometry

#### Detection of immune cells from mouse brain.

Eight to nine weeks old *Il17rc*^*-/-*^ and WT mice were infected with 100 PFU of WNV via f.p. inoculation. The brains were collected after intracardial ice-cold PBS infusion on D7 p.i. and processed into a single-cell suspension using the 40 µm cell strainer. A discontinuous Percoll gradient (GE Healthcare) was used to isolate brain leukocytes after blocking the Fc receptors using 100 µg/ml of rat anti-mouse CD16/CD32 Ig (BD Biosciences) for 30 min. The leukocytes were probed with antibodies against mouse CD45 (PE-Cy7-conjugated, eBioscience, 25-0451-82), CD3 (FITC-conjugated, eBioscience, 11-0031-85 or APC-Cy7-conjugated), CD4 (PerCp Cy 5.5-conjugated, eBioscience, 45-0042-82), CD8 (PE-conjugated, eBioscience, 12-0081-82 or APC-conjugated, eBioscience, 17-0081-82), CD11b (APC-conjugated, BD Biosciences, 561690), and Ly6G surface markers (APC Cy7-conjugated, Invitrogen, A15424). The cells were then fixed with 4% paraformaldehyde (PFA) for 15 min and permeabilized with 1X permeabilization buffer for 30 min. The leukocytes were then probed with antibodies against mouse perforin (PE-conjugated, eBioscience, 12-9392-82) and granzyme B (FITC-conjugated, eBioscience, 11-8898-82). Data were acquired using BD LSRFortessa Cell Analyzer (BD Biosciences) and analyzed with FlowJo (v10.9.0.) software [50].

#### Assessment of immune cell development.

The spleens were collected from eight-week-old *Il17rc*^*-/-*^ and WT mice. The spleen samples were treated with red cell lysis (RBC) buffer and processed into a single-cell suspension using the 70 µm cell strainer. Then, the Fc receptors were blocked with 100 µg/ml of rat anti-mouse CD16/CD32 Ig (BD Biosciences) for 15 min. The splenocytes were stained with antibodies against mouse CD45 (PE-Cy7-conjugated, eBioscience, 25-0451-82), CD3 (FITC-conjugated, eBioscience, 11-0031-85), CD4 (PerCp Cy 5.5-conjugated, eBioscience, 45-0042-82), CD8 (PE-conjugated, eBioscience, 12-0081-82), CD11b (APC-conjugated, BD Biosciences, 561690), and Ly6G surface markers (APC Cy7-conjugated, Invitrogen, A15424). Data were acquired and analyzed as above.

#### Detection of cytotoxic mediators in WNV-specific CD8^+^ T cells.

Eight to nine weeks old *Il17a*^*-/-*^, *Il17rc*^*-/-*^, and WT mice were infected with 20 PFU of WNV via f.p. inoculation. Mice were euthanized on D8 p.i. to collect splenocytes. The splenocytes (5 x 10^6^) were plated in 6-well plates and restimulated with 0.01 MOI of WNV for 24 h at 37°C. During the final 6 h of the stimulation, Brefeldin A solution (BD Biosciences) was added in 1:1000 to block cytokine secretion. To detect the WNV-specific CD8^+^ T cells, splenocytes were incubated with WNV tetramer (NS4B 2488–2496, SSVWNATTA) conjugated with APC (the NIH Tetramer Core Facility at Emory University) and antibody against CD8 (PE-conjugated, eBioscience, 12-0081-82) for 30 min at 4°C in dark. After washing twice with staining buffer, the splenocytes were fixed with 4% PFA for 10 min and permeabilized with 1x permeabilization buffer for 30 min at 4°C. The splenocytes were then stained intracellularly with antibodies against mouse perforin (FITC-conjugated, BioLegend, 154306) and granzyme A (PE-Cy7-conjugated, eBioscience, 25-5831-80) and incubated for 30 min at 4°C in the dark. After washing twice, splenocytes were resuspended in 300µl staining buffer. Data were acquired and analyzed as above.

#### Detection of PI3K-mTOR mediators in CD8^+^ T cells.

Eight to nine weeks old WT mice were infected with 20 PFU of WNV via f.p. inoculation. Mice spleens were collected on D8 p.i. Splenocytes (3 x 10^6^) were plated in 96-well round-bottom plates and probed with methanol-safe surface antibody against mouse CD3 surface marker (FITC-conjugated, eBioscience, 11-0031-85) for 30 min at 4°C in the dark. After washing with RPMI medium once, the splenocytes were treated with 100 ng/ml of mouse rIL-17A. Some rIL-17A-treated wells were also treated with either 12.5 µM of PI3K inhibitor LY294002 (Selleckchem, S1105) or 20 nM of mTOR inhibitor Rapamycin (Selleckchem, S1039) or RPMI medium as a control. The splenocytes were incubated for 1 and 6 h at 37°C in the dark. After incubation, the splenocytes were washed twice with RPMI, fixed with 4% PFA for 15 min, and permeabilized with 90 µl of ice-cold methanol for 30 min at 4°C. Then, the splenocytes were stained with antibodies against mouse CD8 (APC-conjugated, BioLegend, 100712), phospho-S6 (PE-Cy7-conjugated, Invitrogen, 25-9007-42), and phospho-4EBP1 (PE-conjugated, Invitrogen, 12-0107-42) for 30 min at 4°C in the dark. After washing twice, the splenocytes were resuspended in 300 µl staining buffer. Data were acquired and analyzed as above.

The limitation of our flow cytometric analysis is that a Live/Dead stain was not used to differentiate live cells for data analysis due to the limited color choices of the flow cytometer. We acknowledge that some dead cells may be present in our analysis; however, since we applied the same gating strategy for all the samples in the same experiment, this should not affect the overall data trend and the conclusions of the findings.

### Generation of conditional knock-out mice

The *Il17ra*^*fl/fl*^ (JAX stock #031000) male mouse of C57BL/6J background [113], and Cd8a-Cre transgenic (JAX stock #008766) female mouse of C57BL/6N background [114] were purchased from the Jackson Laboratory (Bar Harbor, ME). The *Il17ra*^*fl/fl*^ male and Cd8a-Cre female mice were mated to get the first generation of pups, where 50% of them were *Il17ra*^*fl/w*^ containing the genotype of *fl/w-cre/w.* Then the *Il17ra*^*fl/w*^ mice were backcrossed to *Il17ra*^*fl/fl*^ to get the second generation of pups, where 25% of them were *Il17ra*^*fl/fl*^ containing the genotype of *fl/fl-cre/w* considered as the cre-KO, and the remaining littermates as cre-WT. The total DNA was extracted from blood samples using DNeasy Blood & Tissue Kits (Qiagen) for genotyping by PCR. For the confirmation of cre-KO and cre-WT, targeted DNA sequences were amplified using the primer sequences provided by the Jackson Laboratory.

### Purification of CD8^+^ T cells

Eight to nine-week-old *Il17a*^*-/-*^, *Il17rc*^*-/-*^, and WT mice were infected with 20 PFU of WNV via f.p. inoculation, followed by collection of spleens on D8 p.i. Splenic CD8^+^ T cells were purified from the total splenocytes using a Mouse CD8a^+^ T cell Isolation Kit (Miltenyi Biotec) following the user’s manual.

### RNA sequencing

Library preparation was performed according to the Smart-seq2 protocol [115]. Briefly, cDNA was generated from 50ng of total RNA using SuperScript IV reverse transcriptase (Thermo Fisher Scientific, Cat. #18091050) with oligo(dT) priming and template-switching oligo. Double-stranded cDNA was amplified via 12 cycles of PCR using KAPA HiFi HotStart ReadyMix (Roche) and ISPCR oligo. Amplified cDNA was fragmented using the Nextera XT DNA Library Prep Kit (Illumina), with adapter-ligated products size-selected (200–500 bp) via SPRI beads (Beckman Coulter). Then, libraries were quantified by qPCR (KAPA Biosystems) and pooled at equimolar ratios. Paired-end sequencing (2 × 37 bp) was performed on an Illumina NextSeq 500 platform using a High Output Kit v2 (75 cycles). Raw reads were quality-filtered (FastQC v0.11.9) and aligned to the mm10 with TopHat v2.1.1, Bowtie2 v2.2.8, and Samtools v1.3. Counts were normalized to Fragments per kilobase of exon per million mapped fragments (FPKMs), and differential expression analysis was conducted with Cufflinks v2.2.2. The RNA sequencing data were deposited in Dryad [62].

### CD8^+^ T cell cytotoxicity assay

Eight-week-old *Il17rc*^*-/-*^ and WT mice were infected with 100 PFU of WNV via f.p. inoculation. The brains were collected after intracardial ice-cold PBS infusion on D7 p.i., processed into a single-cell suspension, and treated with RBC lysis buffer. A discontinuous Percoll gradient (GE Healthcare) was used to isolate brain leukocytes. Then, CD8^+^ T cells were purified from the infiltrated leukocytes using a Mouse CD8a^+^ T cell Isolation Kit (Miltenyi Biotec) via negative selection technique. These purified brain-infiltrated CD8^+^ T cells were co-cultured with target cells expressing the ectodomain of WNV-E (MC57GL_WNV-E_) or control cells containing the expression vector (MC57GL_vector_), incubated at 37°C for 8 h at the effector to target ratios of 10:1. After incubation, the cell supernatant was collected and determined the level of cytotoxicity by measuring the release of intracellular LDH using the CyQUANT LDH Cytotoxicity Assay (Invitrogen) following the user’s manual [50,79].

### The *Ex vivo* rIL-17A treatment assay

The isolated splenic CD8^+^ T cells collected from WNV-infected (20 PFU, f.p.) mice were treated with either carrier-free mouse rIL-17A (50 ng/ml, eBioscience, diluted in RPMI medium) or RPMI medium as a control and incubated for 24 h at 37°C. The CD8^+^ T cells were centrifuged at 500 x g for 10 min. After discarding the supernatant, 250 ul of TRI Reagent was added to the cell pellets to conduct the RNA extraction from the CD8^+^ T cells. The gene expression of *PIK3ca*, *mTOR*, and *S6K1* in the rIL-17A-treated or control CD8^+^ T cells was measured by SYBR-based RT-qPCR. The primer sequences used for this study are included in S1 Table.

### Western blot

Eight to nine weeks old *Il17rc*^*-/-*^ and WT mice were infected with 20 PFU of WNV via the f.p. route followed by collecting spleens on D8 p.i. The CD8^+^ T cells were purified from splenocytes and treated with either rIL-17A (100 ng/ml) or RPMI (control) for 15 or 60 min at 37°C. After the treatment, the CD8^+^ T cells were lysed with a mixture containing RIPA lysis buffer, β-ME, and protease and phosphatase cocktail for 5 min. The protein separation was done by gel electrophoresis using 100 µg of total protein in 4–15% sodium dodecyl-sulfate polyacrylamide gel electrophoresis (SDS-PAGE). The proteins were then transferred into a nitrocellulose membrane and probed with primary antibodies against mouse PI3K (p85, 1:500, Cell Signaling Technology, 4257), phosphorated (*p*)-PI3K (p85 [Tyr458]/p55 [Tyr199], 1:200, Cell Signaling Technology, 4228), AKT (1:500, Cell Signaling Technology, 9272), *p*-AKT (Thr308, 1:200, Cell Signaling Technology, 4056), mTOR (1:500, Cell Signaling Technology, 2972), *p*-mTOR (Ser2481, 1:200, Cell Signaling Technology, 2974), pS6K (1:500, Cell Signaling Technology, 9202), *p*-pS6K (Thr389, 1:200, Cell Signaling Technology, 9234), 4EBP1 (1:500, Cell Signaling Technology, 9644), *p*-4EBP1 (Thr37/46, 1:500, Cell Signaling Technology, 2855), and GAPDH as control (1:1000, Cell Signaling Technology, 5174) and incubated overnight at 4°C. The membranes were re-probed with horseradish peroxidase (HRP)-conjugated secondary antibody at RT for 2 h. The protein bands were developed by chemiluminescence substrate (Invitrogen), and images were acquired using the ChemiDoc MP system (Bio-Rad). The densitometric quantification of the protein bands was performed by Image Lab software.

### Metabolomic study

For *in vivo* study, eight to nine weeks old *Il17rc*^*-/-*^ and WT mice were infected with 20 PFU of WNV via f.p. followed by collection of spleens on D8 p.i. CD8^+^ T cells were isolated from splenocytes, snap-frozen by liquid nitrogen, and kept at -80°C until ready for analysis. For *ex vivo* study, the isolated CD8^+^ T cells from WNV-infected WT mice were treated with either carrier-free mouse rIL-17A (100 ng/ml, R&D Systems, diluted in RPMI medium) or RPMI medium as control and incubated for 24 h at 37°C. After washing twice with ice-cold PBS, the CD8^+^ T cells were snap-frozen with liquid nitrogen and kept at -80°C until ready for analysis. The metabolomic assessment of CD8^+^ T cells was performed using liquid chromatography-mass spectrometry (LC-MS) by Gigantest.

### Statistical analysis

Data were compared using the Mann-Whitney U test, log-rank test, or one- or two-tailed Student’s *t*-tests with the GraphPad Prism software (version 10.2.3), whichever was applicable.

## Supporting information

S1 Fig Viral load in different tissues in *Il17rc*^*-/-*^ mice in WNV infection. Eight to nine weeks old *Il17rc*^*-/-*^ (n = 4–8) and WT (n = 4–9) mice were infected with 100 PFU of WNV via the f.p. route. Blood, liver, and spleen tissues were collected, followed by measuring the viral titer by RT-qPCR and expressed as *WNV-E* to mouse *β-actin*. (A) Viral load in blood on D2, 4 p.i. (B) Viral load in liver on D2, 4, 6 p.i. (C) Viral load in spleen on D2, 4, 6 p.i. Data were analyzed using two-tailed Student’s *t*-tests and presented as means.
(TIF)

S2 Fig The antiviral and inflammatory responses in the plasma of Il17rc^-/-^ mice. Eight to nine weeks old *Il17rc*^-/-^ (n = 10) and WT mice (n = 8) were infected with 100 PFU of WNV via f.p. inoculation, plasma was collected on D2 p.i. and the expression of different anti-viral and inflammatory genes was measured by RT-qPCR and normalized to mouse β-actin. The gene expressions of IFN-α (A), IFN-β (B), IFN-γ (C), IL-1β (D), CXCL-2 (E), CXCL-10 (F), and IL-6 (G). Data were analyzed by two-tailed Student’s *t*-tests and presented as mean with ** denotes *p* < 0.01.
(TIF)

S3 Fig Detection of immune cells in spleen and blood in *Il17rc*^*-/-*^ mice. Spleen and blood samples were collected from eight to nine weeks old *Il17rc*^*-/-*^ (n = 3–4) and WT mice (n = 7–10) and characterized by flow cytometry after probing with antibodies against CD45, CD11b, Ly6G, CD3, CD4, and CD8, and measured the cell population. (A-E) Spleen: (A) Neutrophil (CD45^+^CD11b^+^Ly6G^+^); (B) monocyte (CD45^+^CD11b^+^); (C) B cells (CD45^+^CD3^-^CD19^+^); (D) CD4^+^ T cells (CD45^+^CD3^+^CD4^+^); and (E) CD8^+^ T cells (CD45^+^CD3^+^CD8^+^) in *Il17rc*^*-/-*^ and WT mice. (F-J) Blood: (F) Neutrophil (CD45^+^CD11b^+^Ly6G^+^); (G) monocyte (CD45^+^CD11b^+^); (H) B cells (CD45^+^CD3^-^CD19^+^); (I) CD4^+^ T cells (CD45^+^CD3^+^CD4^+^); and (J) CD8^+^ T cells (CD45^+^CD3^+^CD8^+^) in *Il17rc*^*-/-*^ and WT mice Data were analyzed by Mann Whitney U tests *t*-tests and presented as mean ± s.e.m, *p* > 0.05.
(TIF)

S4 Fig The expression of granzyme B in infiltrated CD8^+^ T cells in the brain of *Il17rc*^*-/-*^ mice. Eight to nine weeks old *Il17rc*^*-/-*^ (n = 4) and WT mice (n = 4) were infected with 20 PFU of WNV via f.p. inoculation, and brain tissue was collected on D8 p.i. The brain leukocytes were characterized by flow cytometry after probing with antibodies against CD45, CD3, CD8, and granzyme B. (A) percentages of granzyme B expression in CD8^+^ T cells (CD45^hi^CD3^+^CD8^+^granzyme B^+^); (B) MFI of granzyme B expression in CD8^+^ T cells (CD45^hi^CD3^+^CD8^+^granzyme B ^+^). Data were presented as mean ± s.e.m.
(TIF)

S1 Table Primer sequences used in the study. (DOCX)

## References

[1] Bai F, Thompson EA, Vig PJS, Leis AA. Current understanding of West Nile virus clinical manifestations, immune responses, neuroinvasion, and immunotherapeutic implications. Pathogens. 2019;8(4). 10.3390/pathogens8040193 PMC6963678 31623175

[2] Campbell GL, Marfin AA, Lanciotti RS, Gubler DJ. West Nile virus. The Lancet infectious diseases. 2002;2(9):519–29. 12206968 10.1016/s1473-3099(02)00368-7

[3] Sejvar JJ. West nile virus: an historical overview. The Ochsner Journal. 2003;5(3):6–10. PMC3111838 21765761

[4] Sejvar JJ, Haddad MB, Tierney BC, Campbell GL, Marfin AA, Van Gerpen JA, et al. Neurologic manifestations and outcome of West Nile virus infection. JAMA. 2003;290(4):511–5. doi: 10.1001/jama.290.4.511 12876094

[5] Szatmary G, Leis AA. Concurrent West Nile virus infection in pneumococcal meningitis: clinical and MRI features. J Neuroimaging. 2015;25(2):312–5. doi: 10.1111/jon.12125 24837618

[6] Gould LH, Fikrig E. West Nile virus: a growing concern? J Clin Invest. 2004;113(8):1102–7. doi: 10.1172/JCI21623 15085186 PMC385414

[7] Berner YN, Lang R, Chowers MY. Outcome of West Nile fever in older adults. J Am Geriatr Soc. 2002;50(11):1844–6. doi: 10.1046/j.1532-5415.2002.505402.x 12410904

[8] Smithburn KC, Hughes TP, Burke AW, Paul JH. A Neurotropic Virus Isolated from the Blood of a Native of Uganda. The American Journal of Tropical Medicine. 1940;s1-20(4):471–92. doi: 10.4269/ajtmh.1940.s1-20.471

[9] CDC. Current year data (2024). 2024. [updated January 14, 2025]. https://www.cdc.gov/west-nile-virus/data-maps/current-year-data.html

[10] CDC. Historic Data (1999-2023). [updated February 28, 2025]. https://www.cdc.gov/west-nile-virus/data-maps/historic-data.html

[11] Sejvar JJ. The long-term outcomes of human West Nile virus infection. Clinical infectious diseases: an official publication of the Infectious Diseases Society of America. 2007;44(12):1617–24. 17516407 10.1086/518281

[12] Hadfield J, Brito AF, Swetnam DM, Vogels CBF, Tokarz RE, Andersen KG, et al. Twenty years of West Nile virus spread and evolution in the Americas visualized by Nextstrain. PLoS Pathog. 2019;15(10):e1008042. doi: 10.1371/journal.ppat.1008042 31671157 PMC6822705

[13] Karim SU, Bai F. Introduction to West Nile Virus. West Nile Virus: Methods and Protocols. Springer. 2022. p. 1–7. 10.1007/978-1-0716-2760-0_1 PMC10719965 36331759

[14] Qian F, Wang X, Zhang L, Lin A, Zhao H, Fikrig E, et al. Impaired interferon signaling in dendritic cells from older donors infected in vitro with West Nile virus. J Infect Dis. 2011;203(10):1415–24. doi: 10.1093/infdis/jir048 21398396 PMC3080893

[15] Raychaudhuri SP, Raychaudhuri SK. Mechanistic rationales for targeting interleukin-17A in spondyloarthritis. Arthritis Res Ther. 2017;19(1):51. doi: 10.1186/s13075-017-1249-5 28270233 PMC5341175

[16] Sahu U, Biswas D, Prajapati VK, Singh AK, Samant M, Khare P. Interleukin-17-A multifaceted cytokine in viral infections. J Cell Physiol. 2021;236(12):8000–19. doi: 10.1002/jcp.30471 34133758 PMC8426678

[17] Lubberts E. The IL-23-IL-17 axis in inflammatory arthritis. Nat Rev Rheumatol. 2015;11(7):415–29. doi: 10.1038/nrrheum.2015.53 25907700

[18] Raychaudhuri SP. Role of IL-17 in psoriasis and psoriatic arthritis. Clin Rev Allergy Immunol. 2013;44(2):183–93. doi: 10.1007/s12016-012-8307-1 22362575

[19] Mills KHG. IL-17 and IL-17-producing cells in protection versus pathology. Nature Reviews Immunology. 2022;:1–17. 10.1038/s41577-022-00746-9 PMC9255545 35790881

[20] Toy D, Kugler D, Wolfson M, Bos TV, Gurgel J, Derry J. Cutting edge: interleukin 17 signals through a heteromeric receptor complex. The Journal of Immunology. 2006;177(1):36–9. 16785495 10.4049/jimmunol.177.1.36

[21] Zenobia C, Hajishengallis G. Basic biology and role of interleukin-17 in immunity and inflammation. Periodontol 2000. 2015;69(1):142–59. doi: 10.1111/prd.12083 26252407 PMC4530463

[22] Aggarwal S, Gurney AL. IL-17: prototype member of an emerging cytokine family. J Leukoc Biol. 2002;71(1):1–8. 11781375

[23] Haudenschild D, Moseley T, Rose L, Reddi AH. Soluble and transmembrane isoforms of novel interleukin-17 receptor-like protein by RNA splicing and expression in prostate cancer. J Urol. 2023;23. 10.1074/jbc.M109372200 11706037

[24] McFarland HF, Martin R. Multiple sclerosis: a complicated picture of autoimmunity. Nat Immunol. 2007;8(9):913–9. doi: 10.1038/ni1507 17712344

[25] Zepp J, Wu L, Li X. IL-17 receptor signaling and T helper 17-mediated autoimmune demyelinating disease. Trends Immunol. 2011;32(5):232–9. doi: 10.1016/j.it.2011.02.007 21493143 PMC3329781

[26] Nejati A, Shoja Z, Shahmahmoodi S, Tafakhori A, Mollaei-Kandelous Y, Rezaei F. EBV and vitamin D status in relapsing-remitting multiple sclerosis patients with a unique cytokine signature. Medical Microbiology and Immunology. 2015. 10.1007/s00430-015-0437-7 26365612

[27] Derkow K, Krüger C, Dembny P, Lehnardt S. Microglia Induce Neurotoxic IL-17+ γδ T Cells Dependent on TLR2, TLR4, and TLR9 Activation. PLoS One. 2015;10(8):e0135898. doi: 10.1371/journal.pone.0135898 26288016 PMC4545749

[28] Li D, Guo B, Wu H, Tan L, Chang C, Lu Q. Interleukin-17 in systemic lupus erythematosus: A comprehensive review. Autoimmunity. 2015;48(6):353–61. doi: 10.3109/08916934.2015.1037441 25894789

[29] van den Berg WB, Miossec P. IL-17 as a future therapeutic target for rheumatoid arthritis. Nat Rev Rheumatol. 2009;5(10):549–53. doi: 10.1038/nrrheum.2009.179 19798029

[30] Kim KW, Kim HR, Kim BM, Cho ML, Lee SH. Th17 Cytokines Regulate Osteoclastogenesis in Rheumatoid Arthritis. The American Journal of Pathology. 2015. 10.1016/j.ajpath.2015.07.017 26362732

[31] Gong F, Liu Z, Liu J, Zhou P, Liu Y, Lu X. The paradoxical role of IL-17 in atherosclerosis. Cell Immunol. 2015;297(1):33–9. doi: 10.1016/j.cellimm.2015.05.007 26077826

[32] Lubberts E. The IL-23-IL-17 axis in inflammatory arthritis. Nat Rev Rheumatol. 2015;11(7):415–29. doi: 10.1038/nrrheum.2015.53 25907700

[33] Zenobia C, Hajishengallis G. Basic biology and role of interleukin-17 in immunity and inflammation. Periodontol 2000. 2015;69(1):142–59. doi: 10.1111/prd.12083 26252407 PMC4530463

[34] Raychaudhuri SP. Role of IL-17 in psoriasis and psoriatic arthritis. Clin Rev Allergy Immunol. 2013;44(2):183–93. doi: 10.1007/s12016-012-8307-1 22362575

[35] Chen Y, Qian T, Zhang D, Yan H, Hao F. Clinical efficacy and safety of anti-IL-17 agents for the treatment of patients with psoriasis. Immunotherapy. 2015:1–15. 10.2217/imt.15.50 26055639

[36] Jin B, Zhang Y, Miller HD, He L, Ge D, Wang AR, et al. Defect of IL17 Signaling, but Not Centrinone, Inhibits the Development of Psoriasis and Skin Papilloma in Mouse Models. Biomedicines. 2022;10(8):1976. doi: 10.3390/biomedicines10081976 36009523 PMC9405709

[37] Newcomb DC, Peebles RS Jr. Th17-mediated inflammation in asthma. Curr Opin Immunol. 2013;25(6):755–60. doi: 10.1016/j.coi.2013.08.002 24035139 PMC3855890

[38] Chesne J, Braza F, Mahay G, Brouard S, Aronica M, Magnan A. IL-17 in severe asthma. Where do we stand? American Journal of Respiratory and Critical Care Medicine. 2014;190(10):1094–101. 25162311 10.1164/rccm.201405-0859PP

[39] Strober W, Zhang F, Kitani A, Fuss I, Fichtner-Feigl S. Proinflammatory cytokines underlying the inflammation of Crohn’s disease. Curr Opin Gastroenterol. 2010;26(4):310–7. doi: 10.1097/MOG.0b013e328339d099 20473158 PMC3681421

[40] Siakavellas SI, Bamias G. Role of the IL-23/IL-17 axis in Crohn’s disease. Discov Med. 2012;14(77):253–62. 23114581

[41] Guo N, Shen G, Zhang Y, Moustafa AA, Ge D, You Z. Interleukin-17 Promotes Migration and Invasion of Human Cancer Cells Through Upregulation of MTA1 Expression. Front Oncol. 2019;9:546. doi: 10.3389/fonc.2019.00546 31281798 PMC6596356

[42] Lan R, Zhang K, Niu T, You Z. Genetic alterations of interleukin-17 and related genes in human prostate cancer. Am J Clin Exp Urol. 2019;7(6):352–77. 31970232 PMC6971475

[43] Wang X, Yang L, Huang F, Zhang Q, Liu S, Ma L, et al. Inflammatory cytokines IL-17 and TNF-α up-regulate PD-L1 expression in human prostate and colon cancer cells. Immunol Lett. 2017;184:7–14. doi: 10.1016/j.imlet.2017.02.006 28223102 PMC5362328

[44] Crowe CR, Chen K, Pociask DA, Alcorn JF, Krivich C, Enelow RI, et al. Critical role of IL-17RA in immunopathology of influenza infection. J Immunol. 2009;183(8):5301–10. doi: 10.4049/jimmunol.0900995 19783685 PMC3638739

[45] Mukherjee S, Lindell DM, Berlin AA, Morris SB, Shanley TP, Hershenson MB, et al. IL-17-induced pulmonary pathogenesis during respiratory viral infection and exacerbation of allergic disease. Am J Pathol. 2011;179(1):248–58. doi: 10.1016/j.ajpath.2011.03.003 21703407 PMC3123803

[46] Hou W, Kang HS, Kim BS. Th17 cells enhance viral persistence and inhibit T cell cytotoxicity in a model of chronic virus infection. J Exp Med. 2009;206(2):313–28. doi: 10.1084/jem.20082030 19204109 PMC2646583

[47] Wang Q, Zhou J, Zhang B, Tian Z, Tang J, Zheng Y, et al. Hepatitis B virus induces IL-23 production in antigen presenting cells and causes liver damage via the IL-23/IL-17 axis. PLoS Pathog. 2013;9(6):e1003410. doi: 10.1371/journal.ppat.1003410 23825942 PMC3694858

[48] Neupane B, Acharya D, Nazneen F, Gonzalez-Fernandez G, Flynt AS, Bai F. Interleukin-17A Facilitates Chikungunya Virus Infection by Inhibiting IFN-alpha2 Expression. Front Immunol. 2020;11:588382. 33304351 10.3389/fimmu.2020.588382 PMC7701120

[49] Jie Z, Liang Y, Hou L, Dong C, Iwakura Y, Soong L, et al. Intrahepatic innate lymphoid cells secrete IL-17A and IL-17F that are crucial for T cell priming in viral infection. J Immunol. 2014;192(7):3289–300. doi: 10.4049/jimmunol.1303281 24600029 PMC3967589

[50] Acharya D, Wang P, Paul AM, Dai J, Gate D, Lowery JE, et al. Interleukin-17A Promotes CD8+ T Cell Cytotoxicity To Facilitate West Nile Virus Clearance. J Virol. 2016;91(1):e01529-16. doi: 10.1128/JVI.01529-16 27795421 PMC5165211

[51] Tosello Boari J, Araujo Furlan CL, Fiocca Vernengo F, Rodriguez C, Ramello MC, Amezcua Vesely MC, et al. IL-17RA-Signaling Modulates CD8+ T Cell Survival and Exhaustion During Trypanosoma cruzi Infection. Front Immunol. 2018;9:2347. doi: 10.3389/fimmu.2018.02347 30364284 PMC6193063

[52] Zhang R, Fang H, Chen R, Ochando JC, Ding Y, Xu J. IL-17A Is Critical for CD8+ T Effector Response in Airway Epithelial Injury After Transplantation. Transplantation. 2018;102(12):e483–93. doi: 10.1097/TP.0000000000002452 30211827

[53] Hou L, Jie Z, Desai M, Liang Y, Soong L, Wang T, et al. Early IL-17 production by intrahepatic T cells is important for adaptive immune responses in viral hepatitis. J Immunol. 2013;190(2):621–9. doi: 10.4049/jimmunol.1201970 23233727 PMC3538895

[54] Kohyama S, Ohno S, Isoda A, Moriya O, Belladonna ML, Hayashi H, et al. IL-23 enhances host defense against vaccinia virus infection via a mechanism partly involving IL-17. J Immunol. 2007;179(6):3917–25. doi: 10.4049/jimmunol.179.6.3917 17785829

[55] Liu L, Ge D, Ma L, Mei J, Liu S, Zhang Q, et al. Interleukin-17 and prostaglandin E2 are involved in formation of an M2 macrophage-dominant microenvironment in lung cancer. J Thorac Oncol. 2012;7(7):1091–100. doi: 10.1097/JTO.0b013e3182542752 22534817 PMC3378786

[56] Graham JB, Swarts JL, Lund JM. A Mouse Model of West Nile Virus Infection. Curr Protoc Mouse Biol. 2017;7(4):221–35. doi: 10.1002/cpmo.33 29261232 PMC5777180

[57] Paul AM, Acharya D, Duty L, Thompson EA, Le L, Stokic DS, et al. Osteopontin facilitates West Nile virus neuroinvasion via neutrophil “Trojan horse” transport. Sci Rep. 2017;7(1):4722. doi: 10.1038/s41598-017-04839-7 28680095 PMC5498593

[58] Paul AM, Acharya D, Le L, Wang P, Stokic DS, Leis AA, et al. TLR8 Couples SOCS-1 and Restrains TLR7-Mediated Antiviral Immunity, Exacerbating West Nile Virus Infection in Mice. J Immunol. 2016;197(11):4425–35. doi: 10.4049/jimmunol.1600902 27798161 PMC5123688

[59] Bryan MA, Giordano D, Draves KE, Green R, Gale M, Clark EA. Splenic macrophages are required for protective innate immunity against West Nile virus. PLoS One. 2018;13(2):e0191690. 10.1371/journal.pone.0191690 PMC5800658 29408905

[60] Fan X, Shu P, Wang Y, Ji N, Zhang D. Interactions between neutrophils and T-helper 17 cells. Front Immunol. 2023;14:1279837. doi: 10.3389/fimmu.2023.1279837 37920459 PMC10619153

[61] Colombetti S, Basso V, Mueller DL, Mondino A. Prolonged TCR/CD28 engagement drives IL-2-independent T cell clonal expansion through signaling mediated by the mammalian target of rapamycin. J Immunol. 2006;176(5):2730–8. doi: 10.4049/jimmunol.176.5.2730 16493028

[62] Nazneen F, Neupane B, Chen Y, Karim SU, You Z, Cui W. RNA sequencing data. Dryad; 2025. doi: 10.5061/dryad.2bvq83c14

[63] Gharbi SI, Zvelebil MJ, Shuttleworth SJ, Hancox T, Saghir N, Timms JF, et al. Exploring the specificity of the PI3K family inhibitor LY294002. Biochem J. 2007;404(1):15–21. doi: 10.1042/BJ20061489 17302559 PMC1868829

[64] Breslin EM, White PC, Shore AM, Clement M, Brennan P. LY294002 and rapamycin co-operate to inhibit T-cell proliferation. Br J Pharmacol. 2005;144(6):791–800. doi: 10.1038/sj.bjp.0706061 15778701 PMC1576062

[65] Artemenko M, Zhong SSW, To SKY, Wong AST. p70 S6 kinase as a therapeutic target in cancers: More than just an mTOR effector. Cancer Lett. 2022;535:215593. doi: 10.1016/j.canlet.2022.215593 35176419

[66] Chi H. Regulation and function of mTOR signalling in T cell fate decisions. Nat Rev Immunol. 2012;12(5):325–38. doi: 10.1038/nri3198 22517423 PMC3417069

[67] Kim EH, Suresh M. Role of PI3K/Akt signaling in memory CD8 T cell differentiation. Front Immunol. 2013;4:20. doi: 10.3389/fimmu.2013.00020 23378844 PMC3561661

[68] Bevilacqua A, Li Z, Ho P-C. Metabolic dynamics instruct CD8+ T-cell differentiation and functions. Eur J Immunol. 2022;52(4):541–9. doi: 10.1002/eji.202149486 35253907 PMC9314626

[69] Xiong Y, Lei Q, Zhao S, Guan K. Regulation of glycolysis and gluconeogenesis by acetylation of PKM and PEPCK. In: Cold Spring Harbor symposia on quantitative biology; 2011. 10.1101/sqb.2011.76.010942 PMC4876600 22096030

[70] Ros S, Schulze A. Balancing glycolytic flux: the role of 6-phosphofructo-2-kinase/fructose 2,6-bisphosphatases in cancer metabolism. Cancer Metab. 2013;1(1):8. doi: 10.1186/2049-3002-1-8 24280138 PMC4178209

[71] Fridman A, Saha A, Chan A, Casteel DE, Pilz RB, Boss GR. Cell cycle regulation of purine synthesis by phosphoribosyl pyrophosphate and inorganic phosphate. Biochem J. 2013;454(1):91–9. doi: 10.1042/BJ20130153 23734909

[72] Papandreou C, Li J, Liang L, Bullo M, Zheng Y, Ruiz-Canela M, et al. Metabolites related to purine catabolism and risk of type 2 diabetes incidence; modifying effects of the TCF7L2-rs7903146 polymorphism. Scientific Reports. 2019;9(1):2892. 30814579 10.1038/s41598-019-39441-6 PMC6393542

[73] Reaves ML, Young BD, Hosios AM, Xu Y-F, Rabinowitz JD. Pyrimidine homeostasis is accomplished by directed overflow metabolism. Nature. 2013;500(7461):237–41. 23903661 10.1038/nature12445 PMC4470420

[74] Wang W, Cui J, Ma H, Lu W, Huang J. Targeting Pyrimidine Metabolism in the Era of Precision Cancer Medicine. Front Oncol. 2021;11:684961. doi: 10.3389/fonc.2021.684961 34123854 PMC8194085

[75] Camici M, Garcia-Gil M, Allegrini S, Pesi R, Bernardini G, Micheli V, et al. Inborn Errors of Purine Salvage and Catabolism. Metabolites. 2023;13(7):787. doi: 10.3390/metabo13070787 37512494 PMC10383617

[76] Ortiz GG, Pacheco Moisés FP, Mireles-Ramírez M, Flores-Alvarado LJ, González-Usigli H, Sánchez-González VJ, et al. Oxidative Stress: Love and Hate History in Central Nervous System. Adv Protein Chem Struct Biol. 2017;108:1–31. doi: 10.1016/bs.apcsb.2017.01.003 28427557

[77] Sreekumar PG, Ferrington DA, Kannan R. Glutathione Metabolism and the Novel Role of Mitochondrial GSH in Retinal Degeneration. Antioxidants (Basel). 2021;10(5):661. doi: 10.3390/antiox10050661 33923192 PMC8146950

[78] Runte F, Renner IV P, Hoppe M. Kuby immunology. 2019.

[79] Shrestha B, Diamond MS. Role of CD8+ T cells in control of West Nile virus infection. J Virol. 2004;78(15):8312–21. doi: 10.1128/JVI.78.15.8312-8321.2004 15254203 PMC446114

[80] Shrestha B, Diamond MS. Fas ligand interactions contribute to CD8+ T-cell-mediated control of West Nile virus infection in the central nervous system. J Virol. 2007;81(21):11749–57. doi: 10.1128/JVI.01136-07 17804505 PMC2168805

[81] Shrestha B, Pinto AK, Green S, Bosch I, Diamond MS. CD8 T cells use TRAIL to restrict West Nile virus pathogenesis by controlling infection in neurons. J Virol. 2012;86(17):8937–48. 22740407 10.1128/JVI.00673-12 PMC3416144

[82] Shrestha B, Samuel MA, Diamond MS. CD8+ T cells require perforin to clear West Nile virus from infected neurons. J Virol. 2006;80(1):119–29. doi: 10.1128/JVI.80.1.119-129.2006 16352536 PMC1317548

[83] Engle MJ, Diamond MS. Antibody prophylaxis and therapy against West Nile virus infection in wild-type and immunodeficient mice. J Infect Dis. 2023;83. 10.1128/JVI.77.24.12941-12949.2003 PMC296058 14645550

[84] Neumann H, Medana IM, Bauer J, Lassmann H. Cytotoxic T lymphocytes in autoimmune and degenerative CNS diseases. Trends Neurosci. 2002;25(6):313–9. doi: 10.1016/s0166-2236(02)02154-9 12086750

[85] Sigal LJ. Activation of CD8 T lymphocytes during viral infections. Encyclopedia of immunobiology. 2016. p. 286.

[86] Monin L, Gaffen SL. Interleukin 17 Family Cytokines: Signaling Mechanisms, Biological Activities, and Therapeutic Implications. Cold Spring Harb Perspect Biol. 2018;10(4):a028522. doi: 10.1101/cshperspect.a028522 28620097 PMC5732092

[87] Fredericksen BL. The neuroimmune response to West Nile virus. J Neurovirol. 2014;20(2):113–21. doi: 10.1007/s13365-013-0180-z 23843081 PMC3971464

[88] Getts DR, Terry RL, Getts MT, Müller M, Rana S, Shrestha B, et al. Ly6c+ “inflammatory monocytes” are microglial precursors recruited in a pathogenic manner in West Nile virus encephalitis. J Exp Med. 2008;205(10):2319–37. doi: 10.1084/jem.20080421 18779347 PMC2556789

[89] Bai F, Kong K-F, Dai J, Qian F, Zhang L, Brown CR, et al. A paradoxical role for neutrophils in the pathogenesis of West Nile virus. J Infect Dis. 2010;202(12):1804–12. doi: 10.1086/657416 21050124 PMC3053000

[90] Acheson NH. Fundamentals of molecular virology. John Wiley & Sons. 2011.

[91] Volpe E, Sambucci M, Battistini L, Borsellino G. Fas-Fas Ligand: Checkpoint of T Cell Functions in Multiple Sclerosis. Front Immunol. 2016;7:382. doi: 10.3389/fimmu.2016.00382 27729910 PMC5037862

[92] Castro JE, Listman JA, Jacobson BA, Wang Y, Lopez PA, Ju S, et al. Fas modulation of apoptosis during negative selection of thymocytes. Immunity. 1996;5(6):617–27. doi: 10.1016/s1074-7613(00)80275-7 8986720

[93] Bai F, Town T, Qian F, Wang P, Kamanaka M, Connolly TM. IL-10 signaling blockade controls murine West Nile virus infection. PLoS Pathog. 2009;5(10):e1000610. 10.1371/journal.ppat.1000610 PMC2749443 19816558

[94] Terrell CE, Jordan MB. Perforin deficiency impairs a critical immunoregulatory loop involving murine CD8( ) T cells and dendritic cells. Blood. 2013;121(26):5184–91. 23660960 10.1182/blood-2013-04-495309 PMC3695362

[95] Makedonas G, Hutnick N, Haney D, Amick AC, Gardner J, Cosma G, et al. Perforin and IL-2 upregulation define qualitative differences among highly functional virus-specific human CD8 T cells. PLoS Pathog. 2010;6(3):e1000798. doi: 10.1371/journal.ppat.1000798 20221423 PMC2832688

[96] Janas ML, Groves P, Kienzle N, Kelso A. IL-2 regulates perforin and granzyme gene expression in CD8+ T cells independently of its effects on survival and proliferation. J Immunol. 2005;175(12):8003–10. doi: 10.4049/jimmunol.175.12.8003 16339537

[97] Shacklett BL, Cox CA, Quigley MF, Kreis C, Stollman NH, Jacobson MA, et al. Abundant expression of granzyme A, but not perforin, in granules of CD8+ T cells in GALT: implications for immune control of HIV-1 infection. J Immunol. 2004;173(1):641–8. doi: 10.4049/jimmunol.173.1.641 15210827

[98] Wang H, Xiao Y, Su L, Cui N, Liu D. mTOR Modulates CD8+ T Cell Differentiation in Mice with Invasive Pulmonary Aspergillosis. Open Life Sci. 2018;13:129–36. doi: 10.1515/biol-2018-0018 33817078 PMC7874697

[99] Rex DAB, Dagamajalu S, Gouda MM, Suchitha GP, Chanderasekaran J, Raju R, et al. A comprehensive network map of IL-17A signaling pathway. J Cell Commun Signal. 2023;17(1):209–15. doi: 10.1007/s12079-022-00686-y 35838944 PMC9284958

[100] Cao J, Liao S, Zeng F, Liao Q, Luo G, Zhou YA. Effects of altered glycolysis levels on CD8( ) T cell activation and function. Nat Commun. 2023. 10.1038/s41419-023-05937-3 PMC10329707 37422501

[101] Michalek RD, Gerriets VA, Jacobs SR, Macintyre AN, MacIver NJ, Mason EF, et al. Cutting edge: distinct glycolytic and lipid oxidative metabolic programs are essential for effector and regulatory CD4+ T cell subsets. J Immunol. 2011;186(6):3299–303. doi: 10.4049/jimmunol.1003613 21317389 PMC3198034

[102] Vander Heiden MG, Cantley LC, Thompson CB. Understanding the Warburg effect: the metabolic requirements of cell proliferation. Science. 2009;324(5930):1029–33. doi: 10.1126/science.1160809 19460998 PMC2849637

[103] Guerrini V, Bruiners N, Gennaro ML. Mtorc1 links cellular metabolism and immune functions in Mycobacterium tuberculosis infection and BCG vaccination. The value of BCG and TNF in autoimmunity. Elsevier; 2018. p. 155–70.

[104] Castellano F, Molinier-Frenkel V. Control of T-Cell Activation and Signaling by Amino-Acid Catabolizing Enzymes. Front Cell Dev Biol. 2020;8:613416. doi: 10.3389/fcell.2020.613416 33392202 PMC7773816

[105] Wang W, Zou W. Amino Acids and Their Transporters in T Cell Immunity and Cancer Therapy. Mol Cell. 2020;80(3):384–95. doi: 10.1016/j.molcel.2020.09.006 32997964 PMC7655528

[106] Quéméneur L, Gerland L-M, Flacher M, Ffrench M, Revillard J-P, Genestier L. Differential control of cell cycle, proliferation, and survival of primary T lymphocytes by purine and pyrimidine nucleotides. J Immunol. 2003;170(10):4986–95. doi: 10.4049/jimmunol.170.10.4986 12734342

[107] Soflaee MH, Kesavan R, Sahu U, Tasdogan A, Villa E, Djabari Z, et al. Purine nucleotide depletion prompts cell migration by stimulating the serine synthesis pathway. Nat Commun. 2022;13(1):2698. doi: 10.1038/s41467-022-30362-z 35577785 PMC9110385

[108] Anderson JF, Andreadis TG, Vossbrinck CR, Tirrell S, Wakem EM, French RA, et al. Isolation of West Nile virus from mosquitoes, crows, and a Cooper’s hawk in Connecticut. Science. 1999;286(5448):2331–3. doi: 10.1126/science.286.5448.2331 10600741

[109] Bai F, Wang T, Pal U, Bao F, Gould LH, Fikrig E. Use of RNA interference to prevent lethal murine west nile virus infection. J Infect Dis. 2005;191(7):1148–54. doi: 10.1086/428507 15747251

[110] Neupane B, Bai F. Quantification of West Nile Virus by Plaque-Forming Assay. West Nile Virus: Methods and Protocols. Springer; 2022. p. 9–14. 10.1007/978-1-0716-2760-0_2 PMC10719966 36331760

[111] Chintapula U, Karim SU, Iyer PR, Asokan-Sheeja H, Neupane B, Nazneen F, et al. A novel nanocomposite drug delivery system for SARS-CoV-2 infections. Nanoscale Advances. 2024. 10.1039/d4na00361f PMC11265598 39050946

[112] Nazneen F, Thompson EA, Blackwell C, Bai JS, Huang F, Bai F. An effective live-attenuated Zika vaccine candidate with a modified 5’ untranslated region. NPJ Vaccines. 2023;8(1):50. doi: 10.1038/s41541-023-00650-w 37005424 PMC10066991

[113] Kumar P, Monin L, Castillo P, Elsegeiny W, Horne W, Eddens T, et al. Intestinal Interleukin-17 Receptor Signaling Mediates Reciprocal Control of the Gut Microbiota and Autoimmune Inflammation. Immunity. 2016;44(3):659–71. doi: 10.1016/j.immuni.2016.02.007 26982366 PMC4794750

[114] Maekawa Y, Minato Y, Ishifune C, Kurihara T, Kitamura A, Kojima H, et al. Notch2 integrates signaling by the transcription factors RBP-J and CREB1 to promote T cell cytotoxicity. Nat Immunol. 2008;9(10):1140–7. doi: 10.1038/ni.1649 18724371

[115] Picelli S, Faridani OR, Björklund AK, Winberg G, Sagasser S, Sandberg R. Full-length RNA-seq from single cells using Smart-seq2. Nat Protoc. 2014;9(1):171–81. doi: 10.1038/nprot.2014.006 24385147

