# Supplementary figures and images for: Interleukin-17A signaling promotes CD8+ T cell cytotoxicity against West Nile virus infection through enhancing PI3K-mTOR-mediated metabolism
Source: PLoS Pathog. 2025 Jul 9;21(7):e1013218. doi: 10.1371/journal.ppat.1013218 (PMC12258563; doi:10.1371/journal.ppat.1013218)

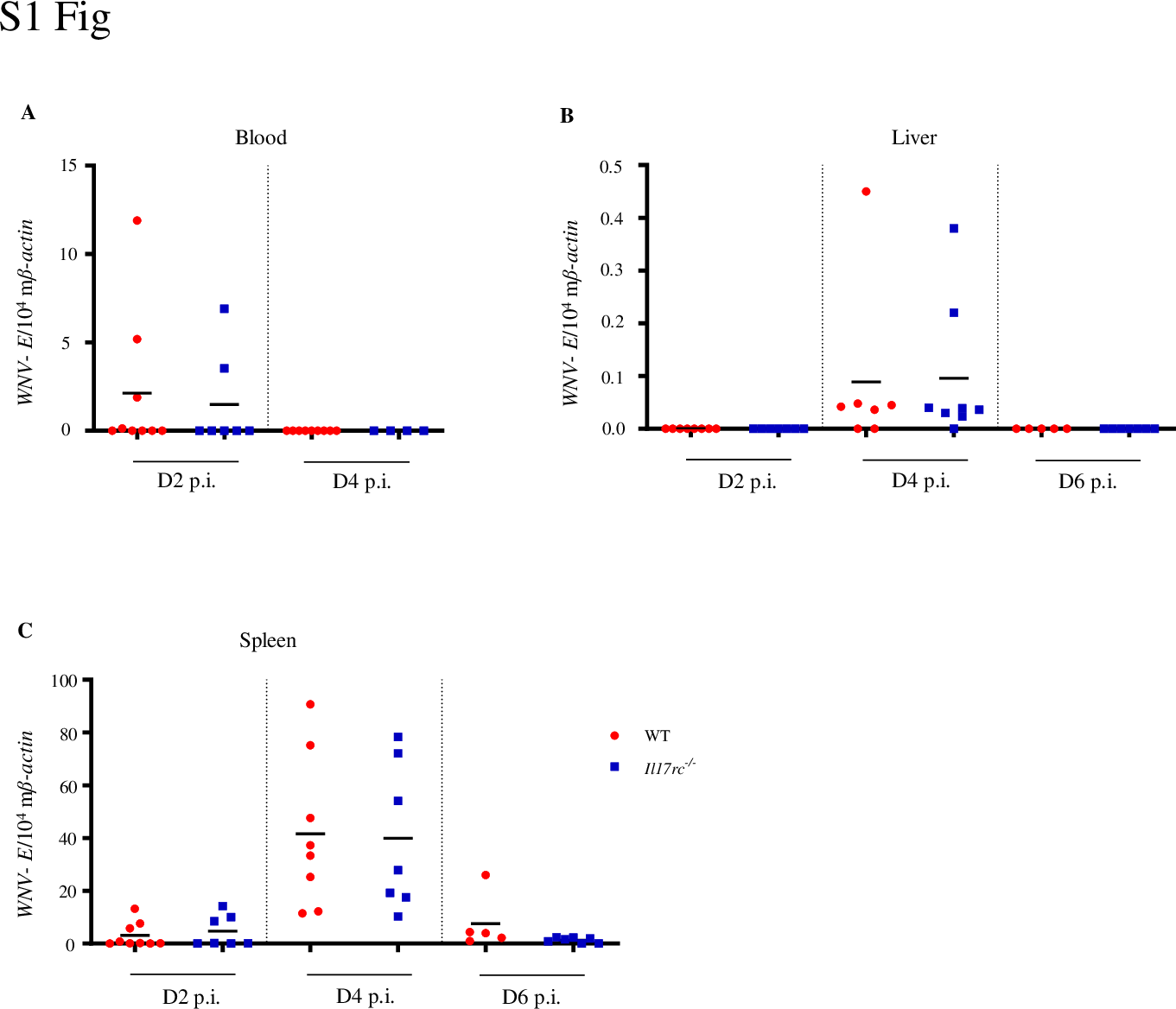

Supplement: S1 Fig — Eight to nine weeks old Il17rc-/- (n = 4–8) and WT (n = 4–9) mice were infected with 100 PFU of WNV via the f.p. route. Blood, liver, and spleen tissues were collected, followed by measuring the viral titer by RT-qPCR and expressed as WNV-E to mouse β-actin. (A) Viral load in blood on D2, 4 p.i. (B) Viral load in liver on D2, 4, 6 p.i. (C) Viral load in spleen on D2, 4, 6 p.i. Data were analyzed using two-tailed Student’s t-tests and presented as means. (TIF) [file ppat.1013218.s001.tif]

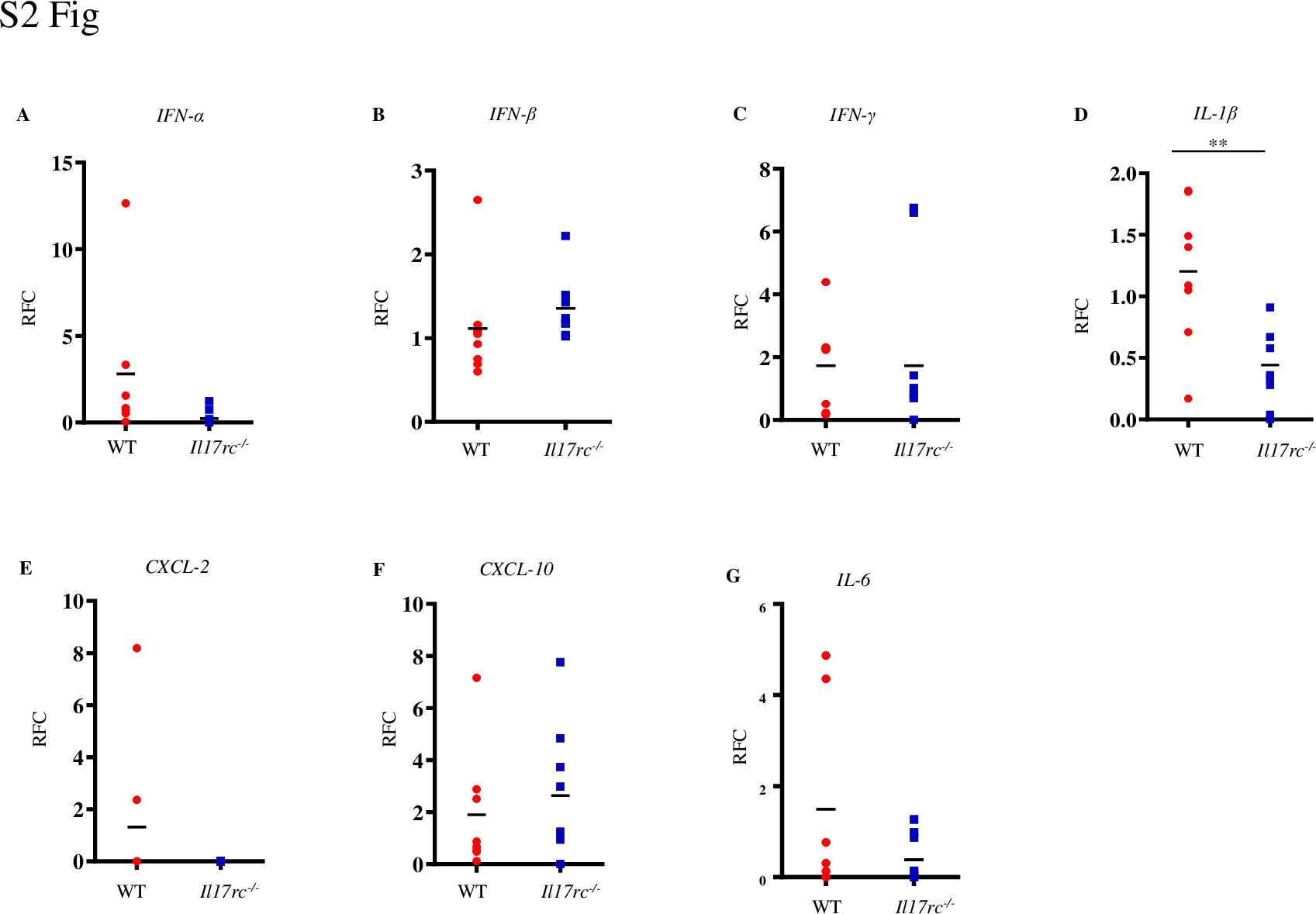

Supplement: S2 Fig — Eight to nine weeks old Il17rc-/- (n = 10) and WT mice (n = 8) were infected with 100 PFU of WNV via f.p. inoculation, plasma was collected on D2 p.i. and the expression of different anti-viral and inflammatory genes was measured by RT-qPCR and normalized to mouse β-actin. The gene expressions of IFN-α (A), IFN-β (B), IFN-γ (C), IL-1β (D), CXCL-2 (E), CXCL-10 (F), and IL-6 (G). Data were analyzed by two-tailed Student’s t-tests and presented as mean with ** denotes p < 0.01. (TIF) [file ppat.1013218.s002.tif]

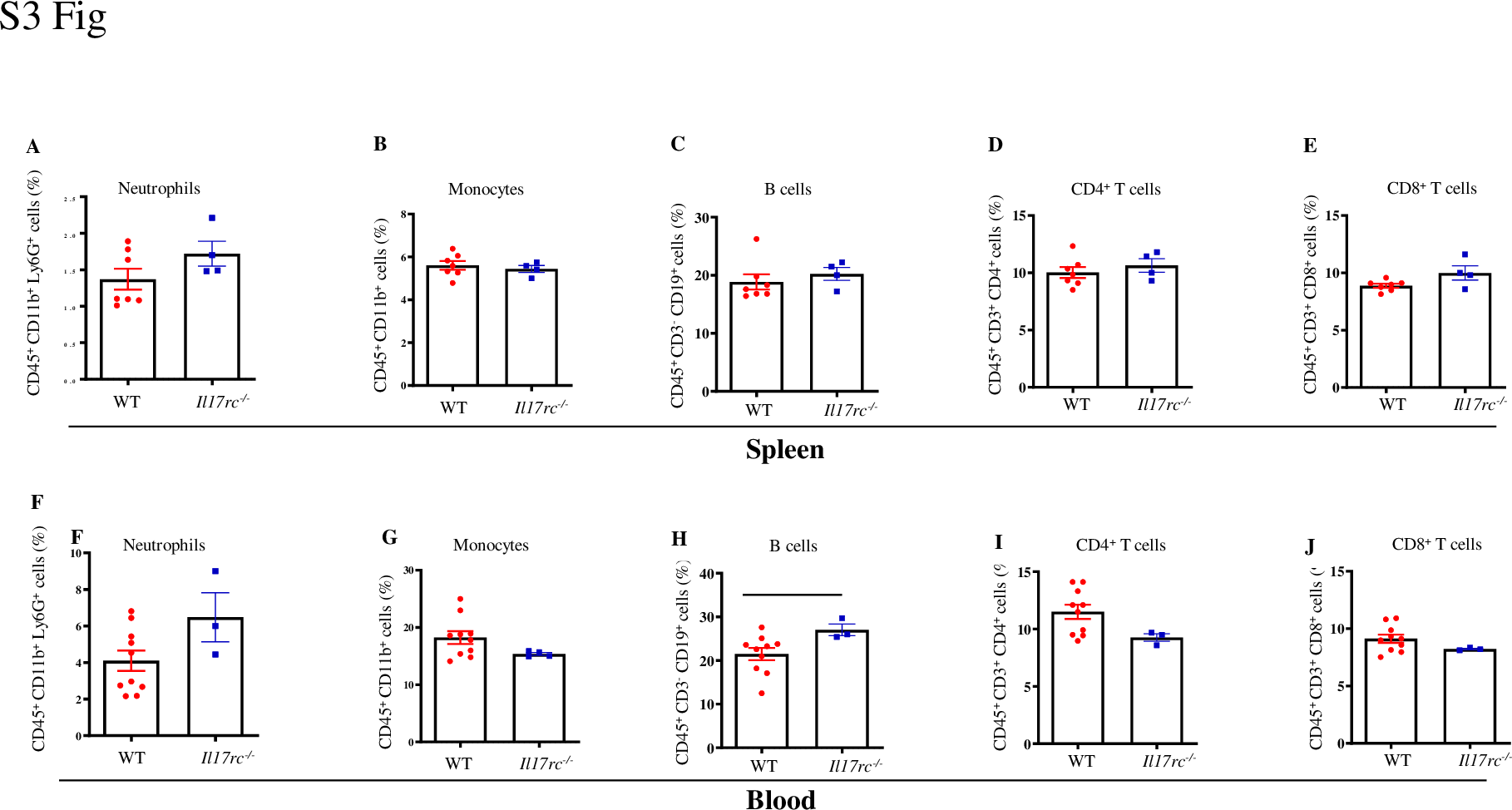

Supplement: S3 Fig — Spleen and blood samples were collected from eight to nine weeks old Il17rc-/- (n = 3–4) and WT mice (n = 7–10) and characterized by flow cytometry after probing with antibodies against CD45, CD11b, Ly6G, CD3, CD4, and CD8, and measured the cell population. (A-E) Spleen: (A) Neutrophil (CD45+CD11b+Ly6G+); (B) monocyte (CD45+CD11b+); (C) B cells (CD45+CD3-CD19+); (D) CD4+ T cells (CD45+CD3+CD4+); and (E) CD8+ T cells (CD45+CD3+CD8+) in Il17rc-/- and WT mice. (F-J) Blood: (F) Neutrophil (CD45+CD11b+Ly6G+); (G) monocyte (CD45+CD11b+); (H) B cells (CD45+CD3-CD19+); (I) CD4+ T cells (CD45+CD3+CD4+); and (J) CD8+ T cells (CD45+CD3+CD8+) in Il17rc-/- and WT mice Data were analyzed by Mann Whitney U tests t-tests and presented as mean ± s.e.m, p > 0.05. (TIF) [file ppat.1013218.s003.tif]

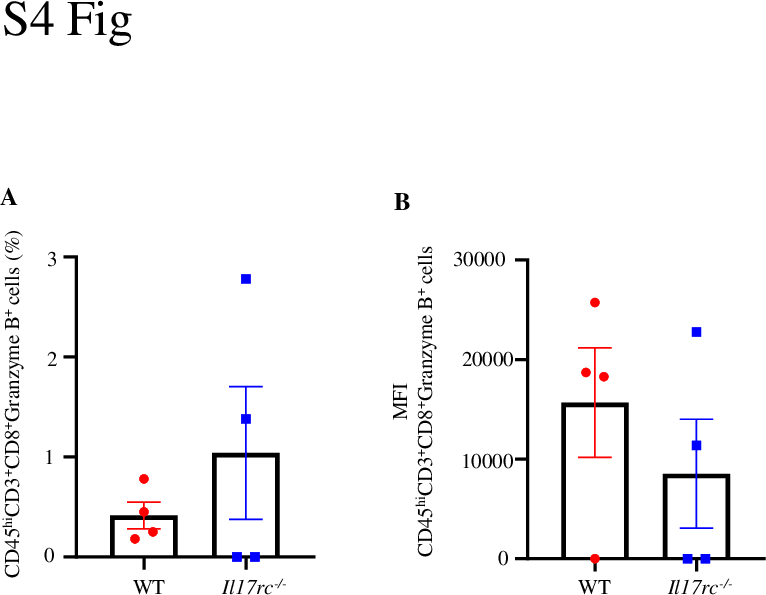

Supplement: S4 Fig — Eight to nine weeks old Il17rc-/- (n = 4) and WT mice (n = 4) were infected with 20 PFU of WNV via f.p. inoculation, and brain tissue was collected on D8 p.i. The brain leukocytes were characterized by flow cytometry after probing with antibodies against CD45, CD3, CD8, and granzyme B. (A) percentages of granzyme B expression in CD8+ T cells (CD45hiCD3+CD8+granzyme B+); (B) MFI of granzyme B expression in CD8+ T cells (CD45hiCD3+CD8+granzyme B +). Data were presented as mean ± s.e.m. (TIF) [file ppat.1013218.s004.tif]
