# Supplementary material for: Interleukin-17A signaling promotes CD8+ T cell cytotoxicity against West Nile virus infection through enhancing PI3K-mTOR-mediated metabolism
Source: PLoS Pathog. 2025 Jul 9;21(7):e1013218. doi: 10.1371/journal.ppat.1013218 (PMC12258563; doi:10.1371/journal.ppat.1013218)
Supplement: S1 Table — (DOCX) [file ppat.1013218.s005.docx]

| Primer | Forward 5′-3′ | Reverse 5′-3′ |
| --- | --- | --- |
| m*β-actin* | AGA GGG AAA TCG TGC GTG AC | CAA TAG TGA TGA TGA CCT GGC CGT |
| *WNV-E* | TTC TCG AAG GCG ACA GCT G | CCG CCT CCA TAT TCA TCA TC |
| m*Il-17a* | TCT CCA CCG CAA TGA AGA CC | TTT CCC TCC GCA TTG ACA CA |
| m*IFN-α* | AGG ACA GGA AGG ATT TTG GA | GCT GCT GAT GGA GGT CAT T |
| m*IFN-β* | CGT TCC TGC TGT GCT TCT CC | TCT TGG AGC TGG AGC TGC TT |
| m*IFN-γ* | CAT TGA AAG CCT AGA AAG TCT G | CTC ATG AAT GCA TCC TTT TTC G |
| m*IL-1β* | TGG TGT GTG ACG TTC CCA TT | CAG CAC GAG GCT TTT TTG TTG |
| m*IL-6* | CGG CCT TCC CTA CTT CAC AA | TCC ACG ATT TCC CAG AGA ACA |
| m*TNF-α* | CAT CTT CTC AAA ATT CGA GTG ACA A | TGG GAG TAG ACA AGG TAC AAC CC |
| m*CXCL-2* | GCG CCC AGA CAG AAG TCA TA | CAG TTA GCC TTG CCT TTG TTC A |
| m*CXCL-10* | GCC GTC ATT TTC TGC CTC A | CGT CCT TGC GAG AGG GAT C |
| m*PI3Kca* | GAA CAA GTA GGC AAC CGT GA | CAG TGC CTC GAG GGA CAA CA |
| *mTOR* | CCA TCC AAT CTG ATG CTG GA | GGT GTG GCA TGT GGT TCT GT |
| m*S6K1* | CCC AAC CCT TCT GAT TTT CA | GAT CTG GGA AGG AGA CAG AA |
